# Mapping Plague Risk Using Super Species Distribution Models and Forecasts for Rodents in the Zhambyl Region, Kazakhstan

**DOI:** 10.1029/2023GH000853

**Published:** 2023-11-13

**Authors:** N. M. Rametov, M. Steiner, N. A. Bizhanova, Z. Zh. Abdel, D. T. Yessimseit, B. Z. Abdeliyev, R. S. Mussagalieva

**Affiliations:** ^1^ National Scientific Center for Particularly Dangerous Infections named after M. Aikimbaev Almaty Kazakhstan; ^2^ Institute of Ionosphere Almaty Kazakhstan; ^3^ Department of Geospatial Engineering Satpaev Kazakh National Research Technical University Almaty Kazakhstan; ^4^ Department of Animal Science Wageningen University and Research Wageningen The Netherlands; ^5^ Laboratory of Theriology Institute of Zoology Almaty Kazakhstan; ^6^ Department of Biodiversity and Bioresources Al‐Farabi Kazakh National University Almaty Kazakhstan; ^7^ Wildlife Without Borders Public Fund Almaty Kazakhstan

**Keywords:** spatial analysis, MaxEnt, climate change, species distribution model, rodents, plague focus

## Abstract

One of the most extensive natural plague centers, or foci, is located in Central Asia, in particular, the Zhambyl region in Southern Kazakhstan. Here, we conducted plague surveillance from 2000 to 2020 in the Zhambyl region in Kazakhstan and confirmed 3,072 cases of infected wild animals. We used Species Distribution Modeling by employing MaxEnt, and identified that the natural plague foci are primarily located in the Moiynqum, Betpaqdala, and Tauqum Deserts. The Zhambyl region's central part, including the Moiynqum and Sarysu districts, has a high potential risk of plague outbreak for the rural towns and villages. Since the phenomenon of climate change has been identified as a determinant that affects the rodent populations, thereby elevating the likelihood of an outbreak of plague, we investigated the potential dissemination routes of the disease under the changing climate conditions, thus creating Species Distribution Forecasts for the rodent species in southern part of Kazakhstan for the year 2100. By 2100, in case of increasing temperatures, the range of host species is likely to expand, leading to a higher risk of plague outbreaks. The highest risk of disease transmission can be expected at the outer limits of the modeled total distribution range, where infection rates are high, but antibody presence is low, making many species susceptible to the pathogen. To mitigate the risk of a potential plague outbreak, it is necessary to implement appropriate sanitary‐epidemiological measures and climate mitigation policies.

## Key Points

- The main plague risk in the Zhambyl comes from four rodent species‐the great gerbil, Libyan jird, midday jird, and yellow ground squirrel
- The distribution of host species suggests that the Moiynqum, Betpaqdala, and Tauqum areas are the natural focal hotspots for plague
- In different climate scenarios, the distribution range of species with plague risk is expected to expand and move north and east

## 1 Introduction

Natural ecosystems have their own specific combination of pathogens, and in the areas of Kazakhstan described in this article, that includes *Yersinia pestis*. In fact, it’s almost universally acknowledged by recent studies that Kazakhstan is the likely origin of the bacterium (Spyrou et al., 2022). The examination of the combination of natural foci for zoonotic pathogens has become increasingly crucial, as it addresses pressing issues in the area of human infectious diseases. This is due to the potential for people to become infected with multiple natural focal pathogens from different causes, resulting in a mixed infection (Demidova et al., 2022).

Serving as reservoir hosts for zoonotic diseases, rodents play a significant role in their transmission and spread in various ways (Dahmana et al., 2020; Douglas et al., 2021; Han et al., 2015; Schmidt et al., 2014; Selmi et al., 2021). As the most numerous and diverse among mammals in the world (Musser, 2022), rodents have been spreading human diseases since the Middle Ages, for example, black rats have been linked to carrying plague (McCormick, 2003). Today, almost 60 different pathogens are transmitted from rodents to humans either directly or indirectly, including the bacterium *Yersinia pestis* which causes a highly infectious disease—plague (Meerburg et al., 2009; Webster, 1996). The rodents carrying these diseases inhabit natural landscapes and can pose a serious threat to the local human population. Recently, several scientific articles showed the interplay between natural sources of disease and the conditions that allow multiple pathogens to circulate in tandem, which is possible due to the existence of a shared parasitic system (Popov et al., 2010; Safronov et al., 2010; Rudakov & Yastrebov, 2015; V. V. Shkarin et al., 2017; Varela et al., 2022).

Currently, the Central Asian desert focus of plague is one of the most active and extensive in the world. Most of it is located in Kazakhstan, covering south and south‐west parts of the country (World Health Organization, 2008). The Central Asian desert focus of plague is broken into small natural foci, mostly located in the Zhambyl region of the southern Kazakhstan,—Betpaqdala, Tauqum, and Moiynqum Deserts being desert natural foci, and Talas Alatau Mountains being high‐mountain focus (Atshabar et al., 2012). It is crucial to carefully monitor the perilous natural focal infections caused by bacteria, viruses, rickettsia, and other etiologies in regions that are prone to plague outbreaks (Kuznetsov et al., 2009; Meka‐Mechenko et al., 2012, 2016; Tynybekov et al., 1997). This is due to the potential threat of an epidemic in these areas, caused by the influence of anthropogenic and climatic factors on the formation of combined natural foci of infectious diseases.

Evidence of an association between climatic factors and plague incidence has been documented in Kazakhstan, that is, temperature increase and rainfall affect the gerbil population and increase plague risk (Linné Kausrud et al., 2007; Stenseth et al., 2006). This highlights the necessity to examine the distribution of the rodents causing most of the plague risk in the current environmental conditions and in the case of climate change. By examining the distribution of these rodents, we attempt to better understand where the disease may be present and where it may spread.

In modern days, Super Species Distribution Models (SuperSDMs) as introduced by Steiner and Huettmann (2023), are the most inclusive and reality‐representative species distribution models possible. They combine open‐access occurrence data of the study species, a large number of well‐descriptive publicly available environmental variables (predictors) and open‐source code, computed by cutting‐edge machine learning algorithms. This approach of Super SDMs allows for much larger input data sets (both for occurrence points and environmental variables) than the conventional SDMs which are mostly based on parsimony, linearity, few predictors and dubious model fittings for probability requiring a strict but unrealistic and rarely achieved research design (MacKenzie et al., 2017; McArdle, 1988).

The Zhambyl region in southern Kazakhstan presents favorable habitat conditions for many rodent species, including the yellow ground squirrel (*Spermophilus fulvus*), great gerbil (*Rhombomys opimus*), small five‐toed jerboa (*Allactaga elater*), common vole (*Microtus arvalis*), Tamarisk jird (*Meriones tamariscinus*), midday jird (*Meriones meridianus*), Libyan jird (*Meriones libycus*), gray dwarf hamster (*Cricetulus migratorius*), wood mouse (*Apodemus sylvaticus*), and house mouse (*Mus musculus*) (Borisenko & Makhmutov, 1977; Fedosenko, 1977; Fedosenko & Borisenko, 1978; Ismagilov, 1969; Kapitonov, 1977; Mokrousov, 1978; Schubin, 1977a). As rodent species are the main plague hosts (Ralle, 1960), their geographical range presents the natural plague foci. Other species that also pose plague risk, the steppe polecat (*Mustela eversmanii*) and the lesser white‐toothed shrew (*Crocidura suaveolens*) are also abundant in this region (Kasabekov & Stogov, 1988; Schubin, 1982a). However, some of the host species, such as the least weasel (*Mustela nivalis*), marbled polecat (*Vormela peregusna*) and the long‐tailed marmot (*Marmota caudata*) occur sporadically (Bekenov, 1982; Kapitonov, 1969; Schubin, 1982b), while others such as the tolai hare (*Lepus tolai*), Eversmann's hamster (*Allocricetulus eversmanni*), and social vole (*Microtus socialis*) are understudied (Baitanaev & Bekenov, 1980; Kapitonov & Borisenko, 1978; Schubin, 1977b). The lack of these data shows the necessity to continue these studies and conduct large‐scale research on desert and semi‐desert species.

In our study, we have several objectives: (a) to examine the combined current distribution of four wild rodent species presenting the highest plague risk—great gerbil (*Rhombomys opimus*), Libyan jird (*Meriones libycus*), midday jird (*Meriones meridianus*), and yellow ground squirrel (*Spermophilus fulvus*) in the Zhambyl region, South Kazakhstan; (b) to predict the future distribution of these species; (c) determine where the areas with the highest plague risk (plague hotspots) are located and discuss the possible scale of plague hotspots in the region for the future under the impact of climate change.

## 2 Materials and Methods

### 2.1 Study Area

**Figure 1:**
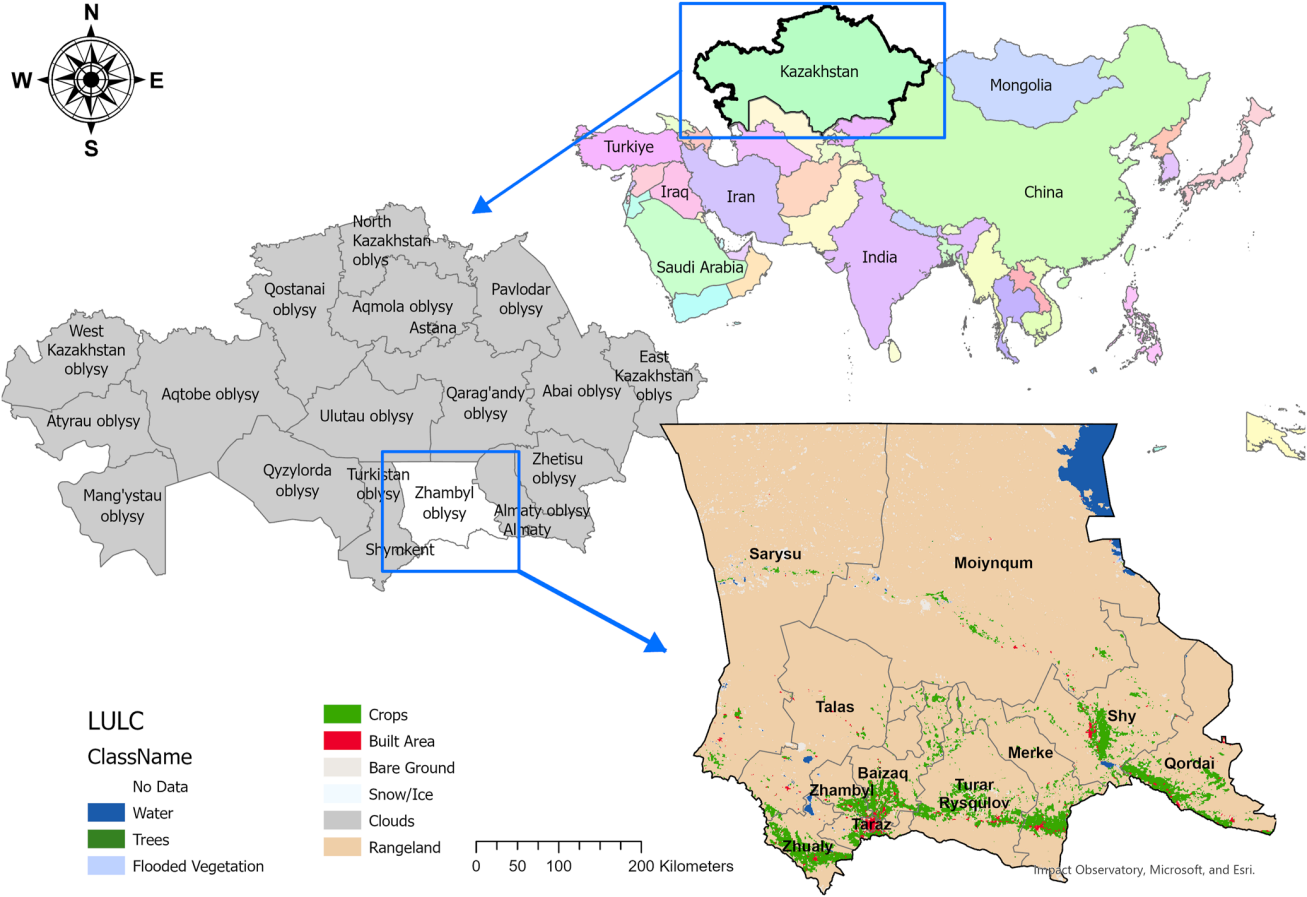
Study area—Zhambyl region, South Kazakhstan.

The study area is located in the Zhambyl region, a southern part of Kazakhstan (see Figure 1). This area covers an extent from 46.1634815°N to 42.2385860°N and from 68.8136057°E to 76.8031342°E, which translates into approximately 144.2 km^2^ (Figure 1). It is located at low altitude (653 m above mean sea level), to the south of the Karatau Mountains, which, in the northwestern direction, gradually decreases and blends into the Qyzylqum and Betpaqdala Deserts (Rybin, 1952). We studied all natural foci within the region: the Betpaqdala, Tauqum, and Moiynqum Deserts, as well as the Talas Alatau Mountains. As most of the natural foci in Kazakhstan are presented by arid zones, our research will focus on the Betpaqdala, Tauqum, and Moiynqum Deserts, and only partially on mountainous areas in Kyrgyz Alatau and Talas Alatau Mountains, which are tendentially less arid.

### 2.2 Data Collection and Pre‐Processing

#### 2.2.1 Plague Surveillance Data

All plague surveillance data used in this study were derived from the plague surveillance database of Aikimbayev's National Scientific Center for Especially Dangerous Infections, a national surveillance network for plague maintained by the Ministry of Health of the Republic of Kazakhstan. According to the general surveillance from 2000 to 2020, 3,072 infected wild animals (14,7701 samples, 2.08% positive) were confirmed by bacteriological tests including microscopy, culture, phage lysis, and mouse inoculation, geocoded by global positioning system (GPS) or detailed address. For the details, see Table S1 in Supporting Information S1. All data layers were projected in EPSG:4326—WGS 84 decimal coordinates and generalized to a pixel resolution of 2.5 × 2.5 km for analysis in QGIS and ArcGIS Pro 3.0 (Environmental Systems Research Institute, Redlands, CA, USA) environment.

The zoological and parasitological study aimed to determine the rodent population through the route‐visual method, compensating for unseen rodents with correction factors (Stepanov et al., 1992). We live‐trapped small mammals (e.g., mouse‐like rodents) with Gero snap‐traps equipped with bait and captured medium‐sized mammals with arc fenn traps (kapkans) or live traps (e.g., Sherman, Zaycev's live traps) (Mamedzade et al., 2019). Specimens were collected for examination in the laboratory. We collected and divided ectoparasites into groups for research, with the presence of plague tested in a sample of 25–30 fleas or mites (Guidelines for emergency field and settlement prevention of plague, 1998). We studied and suspended mites separately in a saline solution before inoculation for bacteriological analysis. The isolated plague microbe was identified through various standard microbiological and biochemical methods, with the microbe isolated by infecting laboratory rodents with internal organs (Nekrasova et al., 2001, 2009). The RNGA‐RNAt and RNHA‐RNAg serological reactions and classical and real‐time PCR methods were used to identify the isolated strains of the plague microbe (Engelthaler et al., 1999; Nyirenda et al., 2017).

#### 2.2.2 Species Distribution Models (SDMs) Created With MaxEnt

For this study, we created the combined species distribution models (SDMs), for which we utilized the Open‐Access Machine Learning software MaxEnt vers. 3.4.4 (Merow et al., 2013). For the model runs, we have utilized the default settings for all SDMs. These settings entail “Auto Features” for the selection of the applied features, 500 maximum iterations, and all additional settings kept on default. In order to assess the fit of the models and the top predictors for each model, we obtained Area Under the Curve (AUC) indices and the three top predictors from the HTML model output and added them to each model in the discussion section. Overall, these metrics are not our main focus in evaluating the models; it is rather the predictions themselves (“let the models speak for themselves”—*sensu* Breiman, 2001). A more detailed overview of all top predictors for each model can be found in Table S2 in Supporting Information S1. We divided occurrence records into two categories—Records with positive antibodies (AB‐positive records) and Total plague risk records (Combined culture and AB‐positive records).

**Figure 2:**
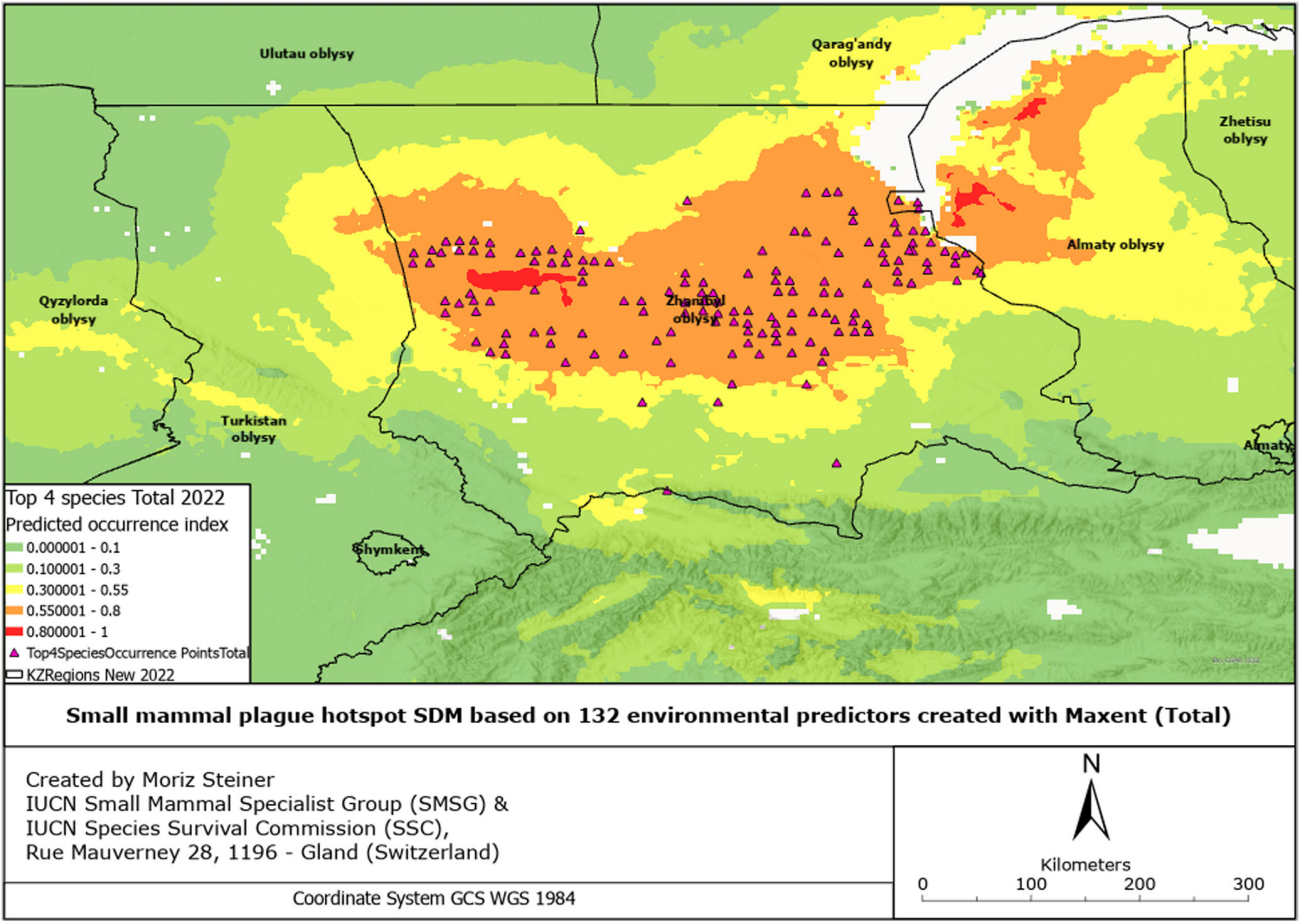
Rodent plague hotspot SDM based on 132 environmental predictors (Total).

**Figure 3:**
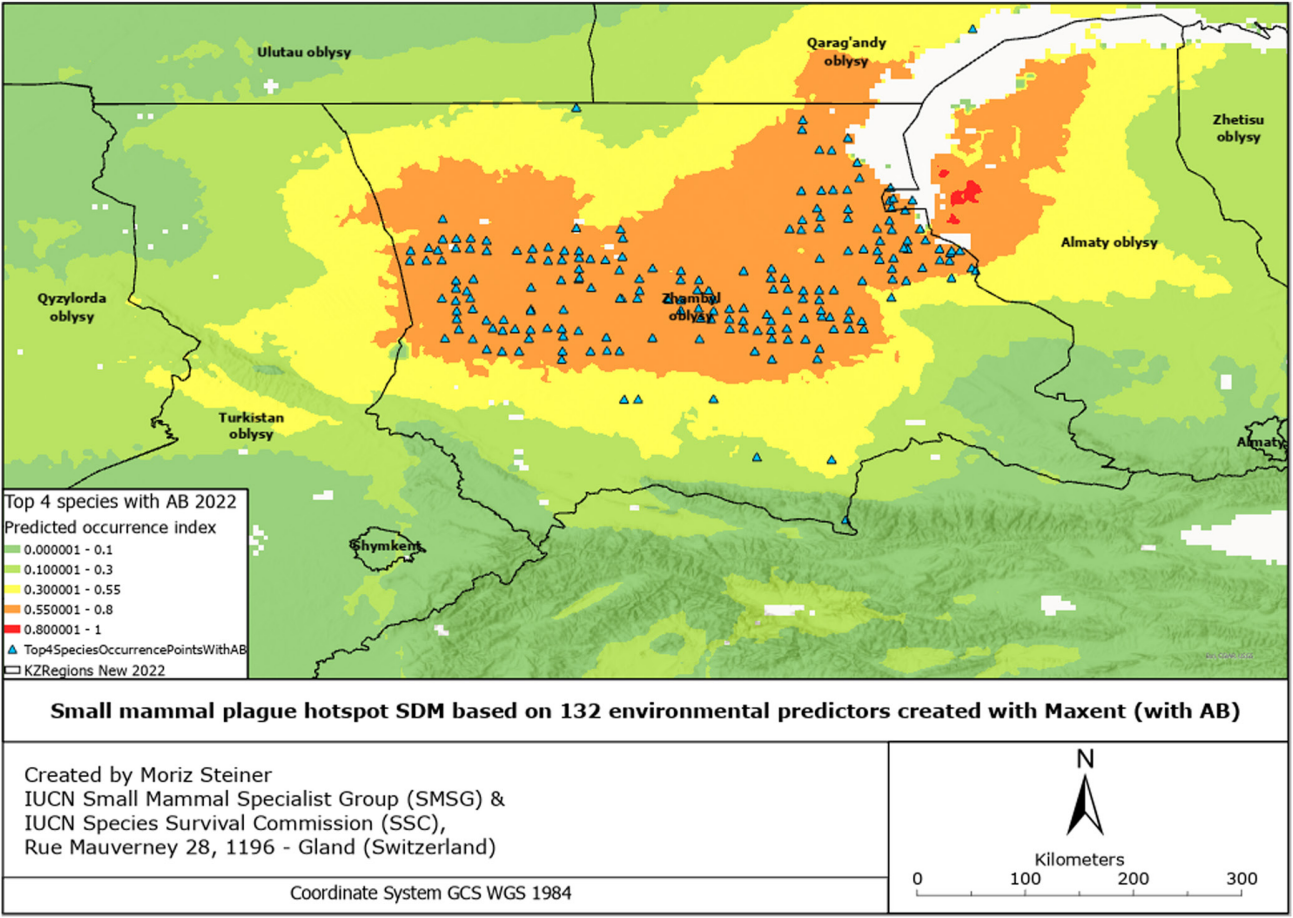
Rodent plague hotspot SDM based on 132 environmental predictors (with positive AB records).

**Figure 4:**
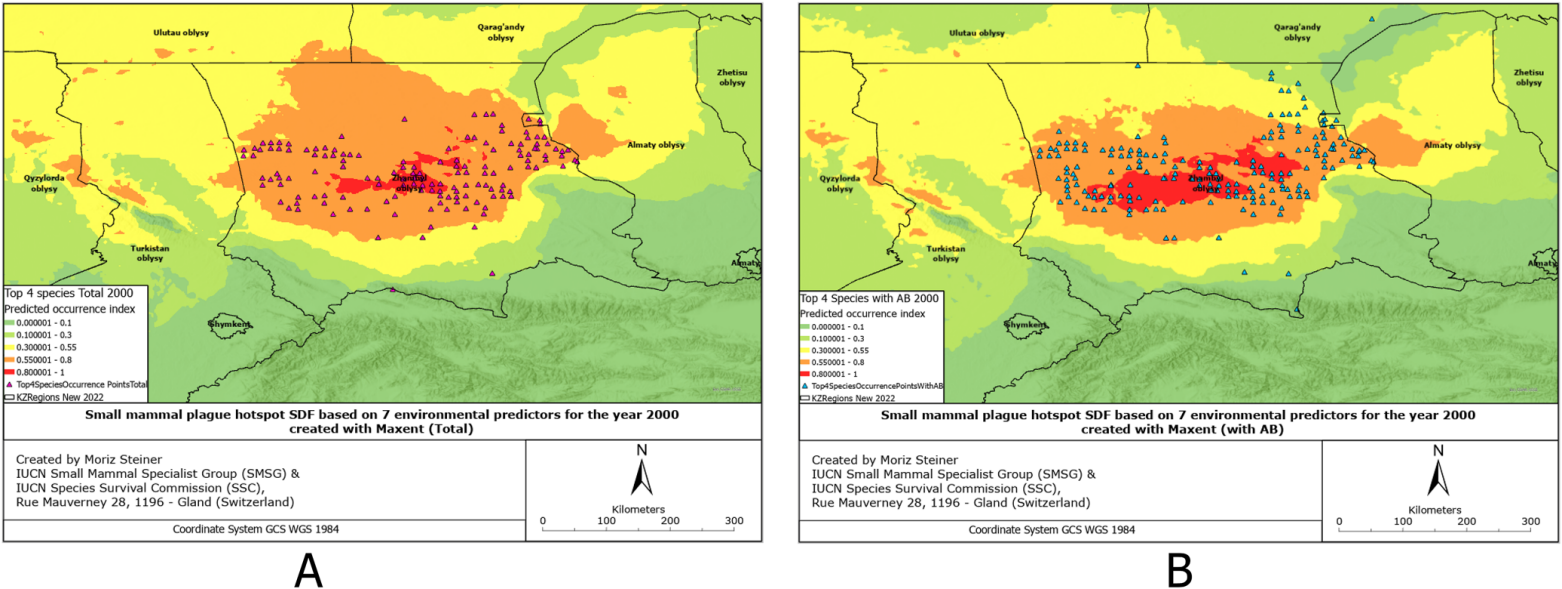
Rodent plague risk hotspot SDF based on 7 environmental predictors for the year 2000 created with MaxEnt (a) Total, (b) with AB.

**Figure 5:**
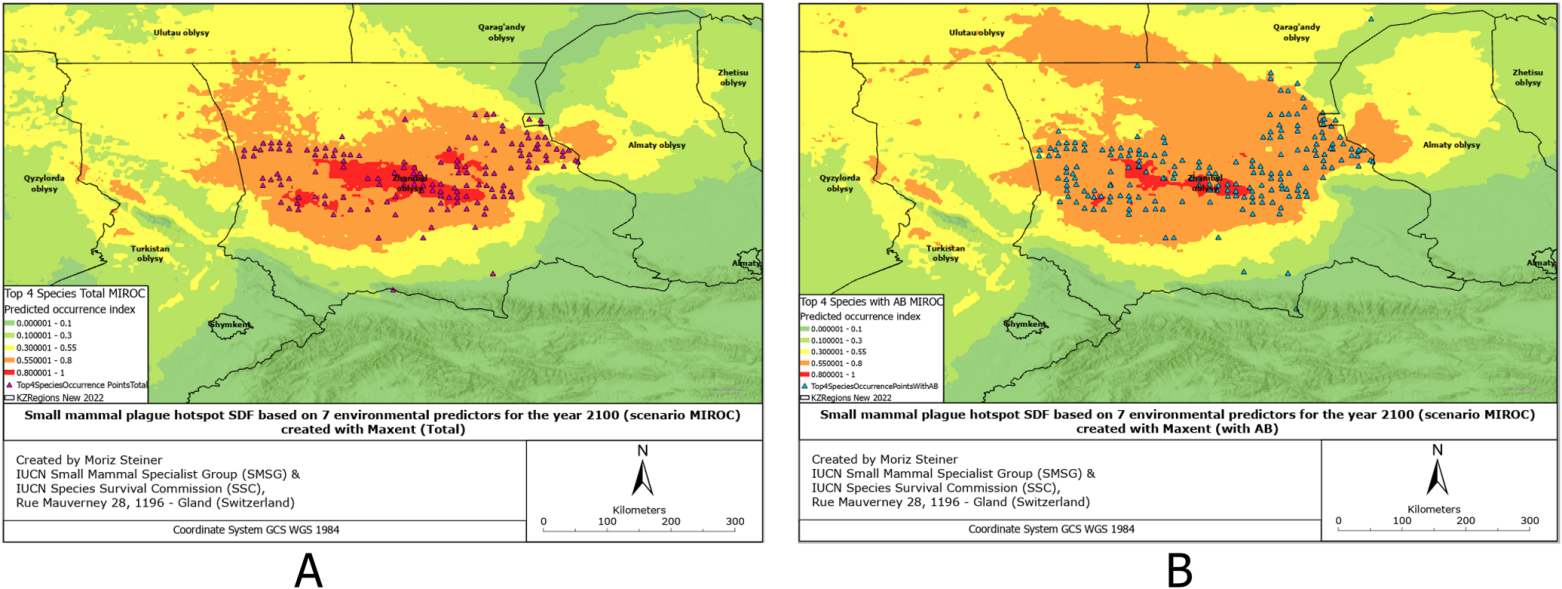
Rodent plague risk hotspot SDF based on 7 environmental predictors for the year 2100 (scenario MIROC) created with MaxEnt (a) Total, (b) with AB.

**Figure 6:**
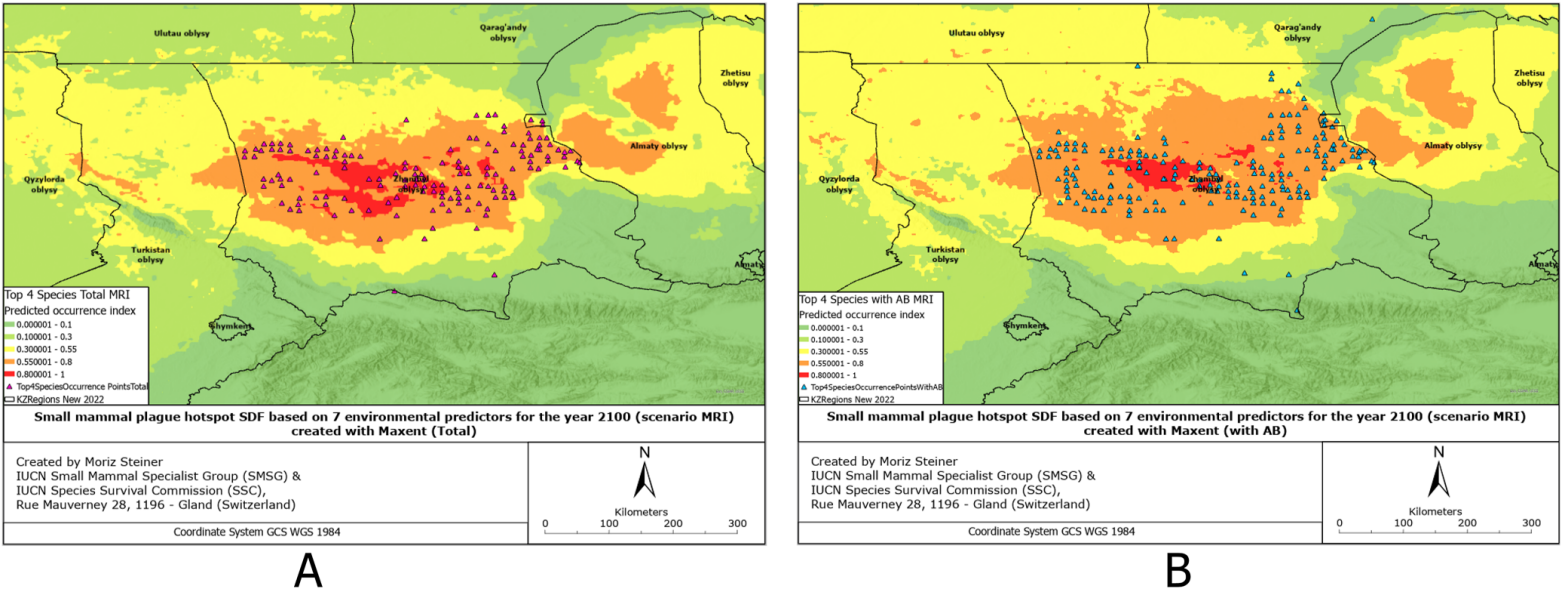
Rodent plague risk hotspot SDF based on 7 environmental predictors for the year 2100 (scenario MRI) created with MaxEnt (a) Total, (b) with AB.

**Figure 7:**
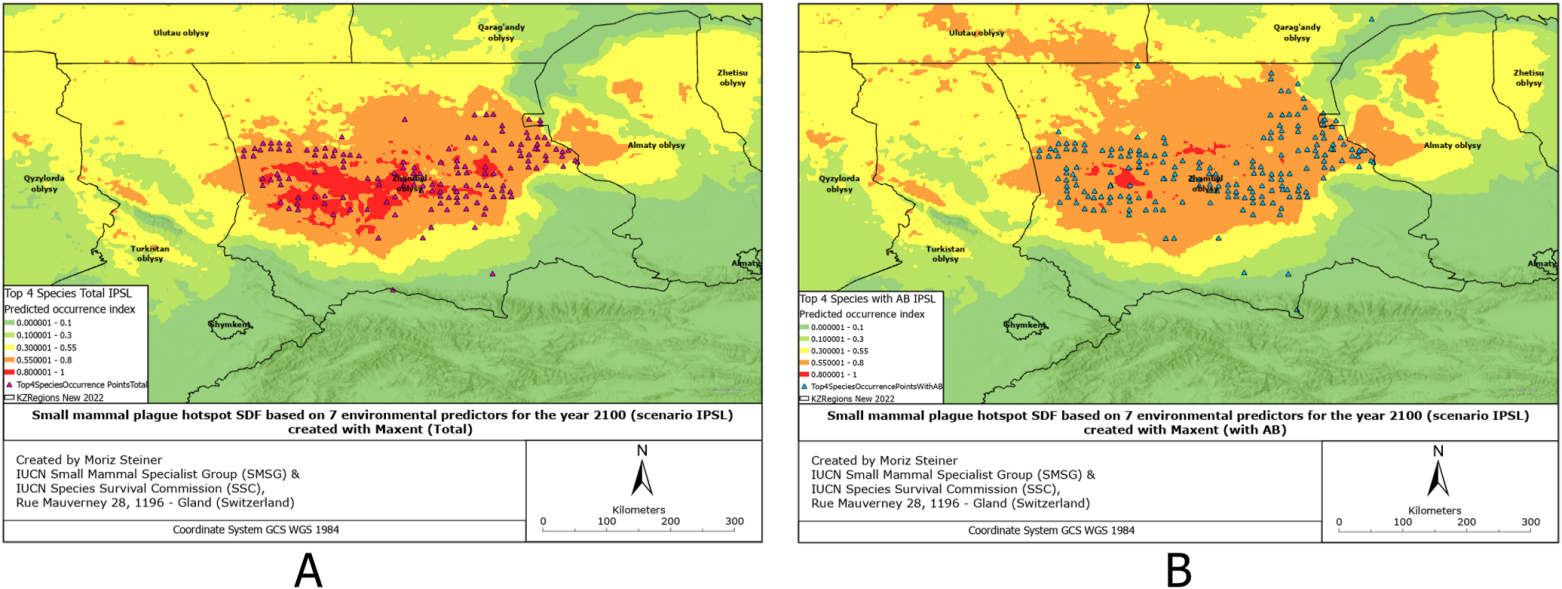
Rodent plague risk hotspot SDF based on 7 environmental predictors for the year 2100 (scenario IPSL) created with MaxEnt (a) Total, (b) with AB.

SDMs were created based on 132 environmental predictors, arguably also called Super SDMs (see Steiner & Huettmann, 2023). Additionally, we combined all the occurrence points from all species of small mammals from which we had records (Figures S1–S6 in Supporting Information S1). These SDMs have also been created for all observed species individually (Figures S7–S17 in Supporting Information S1), and combined for four rodent species to provide insight into the overall plague risk (Figures 2, 3, 4, 5, 6, 7). All SDMs have been created for the total observations and in more detail also for AB‐present occurrences separately. SDMs for the four rodent species have been created individually (Figures S7–S10 in Supporting Information S1), and combined their occurrences to provide insight into the overall plague risk (Figures 2, 3, 4, 5, 6, 7).

The high number of included environmental predictors aims to best describe the surrounding environment and habitat conditions of the included species (see examples of studies with high predictor numbers such as Bizhanova et al., 2022; Steiner & Huettmann, 2023. These are the building blocks of the SDMs, apart from the collected occurrence data (Lissovsky & Dudov, 2021). These predictors include factors such as temperature, precipitation, relative humidity, soil conditions, snowfall, proximity to streets, cities, rivers, wildfires, vegetation cover, climate classes, prey densities, etc. on a global scale. Further information on the predictors, including their sources, can be found in Bizhanova et al. (2022) and Steiner and Huettmann (2023). Each environmental predictor has an accuracy of 2.5 km^2^ with a pixel size of 0.04166666666666666435 × −0.04166666666666666435 decimal degrees (CRS: EPSG:4326–WGS 84) and is considered to be the most comprehensive set of environmental predictors that is publicly available to date. Methods used for standardizing and preparing the predictors for use in SDMs, can be found in Steiner and Huettmann (2023), specifically Chapter 3.

We created the combined SDMs based on the occurrence points of the great gerbil (*Rhombomys opimus*), Libyan jird (*Meriones libycus*), midday jird (*Meriones meridianus*) and yellow ground squirrel (*Spermophilus fulvus*) in the Zhambyl region, Kazakhstan. These species have been chosen for SDMs from a list of species for which field data has been collected, as they presented the highest plague risk and had the most records (allowing for more reliable SDMs). Additionally, we created SDMs for other species of wild small mammals from orders Eulipotyphla, Carnivora, Lagomorpha, and Rodentia from which we had data. These models were created individually and once with all species combined in order to provide an overview of the total small mammal plague risk in the Zhambyl region. The complete list of all included species can be observed in Table S3 in Supporting Information S1, and the combined and species‐specific SDMs for all species not included in the main text can be found in Figures S1–S17 in Supporting Information S1.

As seen in Table S3 in Supporting Information S1, we included four of the above‐mentioned species there; and included the individual SDMs of other species in Figures S7–S17 in Supporting Information S1, while the rest instead had to be discarded due to insufficient data quantities. This selection was based on the higher plague risk and availability of occurrence data from the sample species. MaxEnt needs at least two different occurrence locations per “species” to successfully create a SDM. If there were fewer than two different occurrence locations, then the SDM for the corresponding species was discarded. For species for which no AB‐positive occurrence points were recorded, we still created SDMs—for completion—and possible future research projects. These latter‐discussed SDMs have therefore been illustrated in Figures S7–S17 in Supporting Information S1.

Combined SDMs of the rodent species with the highest number of records and the greatest potential for plague risk can be found in Section 3.

#### 2.2.3 Species Distribution Forecasts (SDFs) Created With MaxEnt

In order to gain insight into the potential risk of a rodent plague in the future, we conducted a Species Distribution Forecasts (SDFs) for the year 2100. These SDFs were generated using the MaxEnt software, with the same parameters as the previously mentioned Species Distribution Models (SDMs). However, we used a different set of predictors, namely seven bioclimatic variables for the forecast. We incorporated three distinct scenarios (MIROC, MRI, and IPSL) to account for varying climate conditions in the future. To compare the results from the year 2100 with the current state (from the year 2000), we also applied these seven environmental predictors to the year 2000. The reason for only utilizing seven BioClim layers in this forecast analysis (compared to the 132 environmental predictors utilized earlier) is that most variables are not available for the year 2100 (e.g., position of roads, rivers, or tree cover, etc.). Therefore, with the best‐available publicly available variables, only a small set of environmental variables was able to be used.

These three scenarios aim to account for the various climate changes that are expected to occur by 2100. The scenarios used in this study are Global Climate Models (GCMs) that we obtained from WorldClim.org (Fick & Hijmans, 2017) and include MIROC6 (approx. RPC 2.6), MRI‐ESM2‐0 (approx. RPC 4.5), and IPSL‐CM6A‐LR (approx. RPC 6) (Nexus, 2023). The MIROC6 model represents a scenario with a low temperature increase or even a cooling scenario (Tatebe et al., 2019). The MRI‐ESM2‐0 model represents a low to medium temperature increase, with an estimated global increase of 2 degrees Celsius (Yukimoto et al., 2019). This scenario can be viewed as the model with the highest probability of occurring. Lastly, the IPSL‐CM6A‐LR model represents a medium to high temperature increase, with an estimated increase of approximately 3 degrees Celsius (Boucher et al., 2020).

## 3 Results

### 3.1 Species Distribution Models

We created Species Distribution Models (SDMs) using 132 environmental predictors and a total of 3,075 species occurrence points. To provide an overview of the total distribution of rodents and the plague‐infected range in Kazakhstan, we created SDMs for those two groups (total and with AB—see Figures 2 and 3 accordingly). For more sophisticated insights, these SDMs have also been created including all the other species of small mammals and for each species individually (see Figures S1–S17 in Supporting Information S1). Table S3 in Supporting Information S1 shows the species for which too few occurrence locations were available to create the SDMs, indicated as “Discarded.” For some of the species, there were no locations recorded with a positive antibody (AB) record. For those species, we still created SMDs that can be used for further research and other studies (see Figures S7–S17 in Supporting Information S1). For the remaining species, there were positive AB records available with enough occurrence locations, which have also been included in Figures S7–S17 in Supporting Information S1.

Figure 2 shows the distribution of four rodent species considered for this research, combining all general sightings of these mammals without antibody (AB) records. Thus, the model illustrates the total plague risk hotspot in the southern parts of Kazakhstan, specifically in the Zhambyl and Almaty regions. We can observe that the central part of the Zhambyl region and the north‐western part of the Almaty region along Balkhash Lake show an increased plague risk compared to the southernmost areas of these regions.

Figure 3 illustrates the plague risk hotspots of considered rodents with observed antibodies (AB) in the southern parts of Kazakhstan. Similarly, to the first model, this model indicates the high occurrence of the species with AB records in most of the Zhambyl region's districts, predominately in its central area, as well as the north‐western part of the Almaty region, and the southern part of the Qaraghandy region.

### 3.2 Species‐Specific SDMs

Distribution models for each specific species of small mammals (SDMs) are shown in Figures S7–S15 in Supporting Information S1.

Figure S7a in Supporting Information S1 represents the total distribution of PCR‐positive great gerbil (*Rhombomys opimus*), showing one major hotspot region in the southern part of central Kazakhstan with more condensed hotspots on the southwest coast of Lake Balkhash and around Zhaylaukol. The AUC for this model was 0.995 with the top three predictors being World Soil Characteristics, Bio 7 (Temperature Annual Range (BIO5‐BIO6)), and Maximum Temperature in February 2020. Figure S7b in Supporting Information S1 represents the distribution of ELISA AB‐positive great gerbil individuals. Here the distribution range is similar to the one for Figure S7a in Supporting Information S1, but with no significant condensed hotpots. The AUC for this model was 0.990 with the top three predictors being World Soil Characteristics, Bio 7, and Maximum Temperature in February 2020.

According to the SDM of the yellow ground squirrel (*Spermophilus fulvus*), shown in Figure S8a in Supporting Information S1, we can see one major hotspot in the southern part of central Kazakhstan. The AUC for this model was 0.998 with the top three predictors being World Soil Characteristics, Maximum Relative Humidity in October 2020, and Maximum Relative Humidity in September 2020. Figure S8b in Supporting Information S1 represents the distribution of AB‐positive yellow ground squirrel individuals. Here there are no major hotspots visible, but rather a scattered distribution of very small hotspots throughout the southeastern part of Kazakhstan and some patches in southeastern Kyrgyzstan. The AUC for this model was 1,000 with the top three predictors being World Soil Characteristics, Proximity to the World's Protected Areas, and Bio 9.

The SDM of Libyan jird (*Meriones libycus*) without AB records (based on general occurrence points), illustrated in Figure S9a in Supporting Information S1, suggests one major hotspot in southern Kazakhstan, subdivided into three hotpots close to Bayqadam, Alekseyevka, and the southeastern coast of Balkhash Lake (all located in Kazakhstan). The AUC for this model was 0.997 with the top three predictors being World Soil Characteristics, Maximum Relative Humidity in October 2020, and Maximum Relative Humidity in September 2020. Figure S9b in Supporting Information S1 represents the distribution of AB‐positive Libyan jird individuals. Here the most significant modeled distribution covers again the southern part of central Kazakhstan, with hotspots in the areas surrounding Qaraboget, Qaraoi, and Baqyrly (all located in Kazakhstan). The AUC for this model was 1,000 with the top three predictors being World Soil Characteristics, Maximum Relative Humidity in October 2020, and Bio 9 (Mean Temperature of Driest Quarter).

Figure S10a in Supporting Information S1 represents the total distribution of midday jird (*Meriones meridianus*), showing similar distribution hotspots to the ones described in Figure S9a in Supporting Information S1. The AUC for this model was 0.997 with the top three predictors being World Soil Characteristics, Maximum Relative Humidity in October 2020, and Maximum Relative Humidity in September 2020. Similarly, Figure S10b in Supporting Information S1 shows the distribution of AB‐positive midday jird individuals. Here again, the hotspots are close to the ones described in Figure S9b in Supporting Information S1, except for the central hotpot which is in this model located a bit further south. The AUC for this model was 1,000 with the top three predictors being World Soil Characteristics, Maximum Relative Humidity in October 2020, and Bio 9.

SDMs were also created for the other 5 small mammals found in South Kazakhstan: small five‐toed jerboa (*Allactaga elater*), wood mouse (*Apodemus sylvaticus*), common vole (*Microtus arvalis*), steppe polecat (*Mustela eversmanii*), and shrews (*Soricidae*) (all found in Figure S11–S15 in Supporting Information S1). The models were based on the presence of AB‐positive individuals. The models indicate that there are various hotspots of these species, with the locations of hotspots differing depending on the species and the presence of AB‐positive individuals. The total distribution of small five‐toed jerboas is shown to have three hotspots in the vicinity of Zhaylaukol, Qaraboget, and Bogodukhovka in Kazakhstan. The distribution of AB‐positive small five‐toed jerboa individuals shows a fragmented distribution range with no clear hotspots. The SDM of wood mice (total records) shows a major hotspot in northeast Kyrgyzstan, while the hotspots of AB‐positive individuals shifted northwards. The SDM of common voles and steppe polecats also indicate hotspots in northeast Kyrgyzstan. The top three predictors in each model were different but included World Soil Characteristics, temperature & humidity, slope, proximity to major lakes, forest fires, vegetation cover, and proximity to protected areas. All models had an AUC score of 1,000.

### 3.3 Species Distribution Forecasts (SDFs)

**Figure 8:**
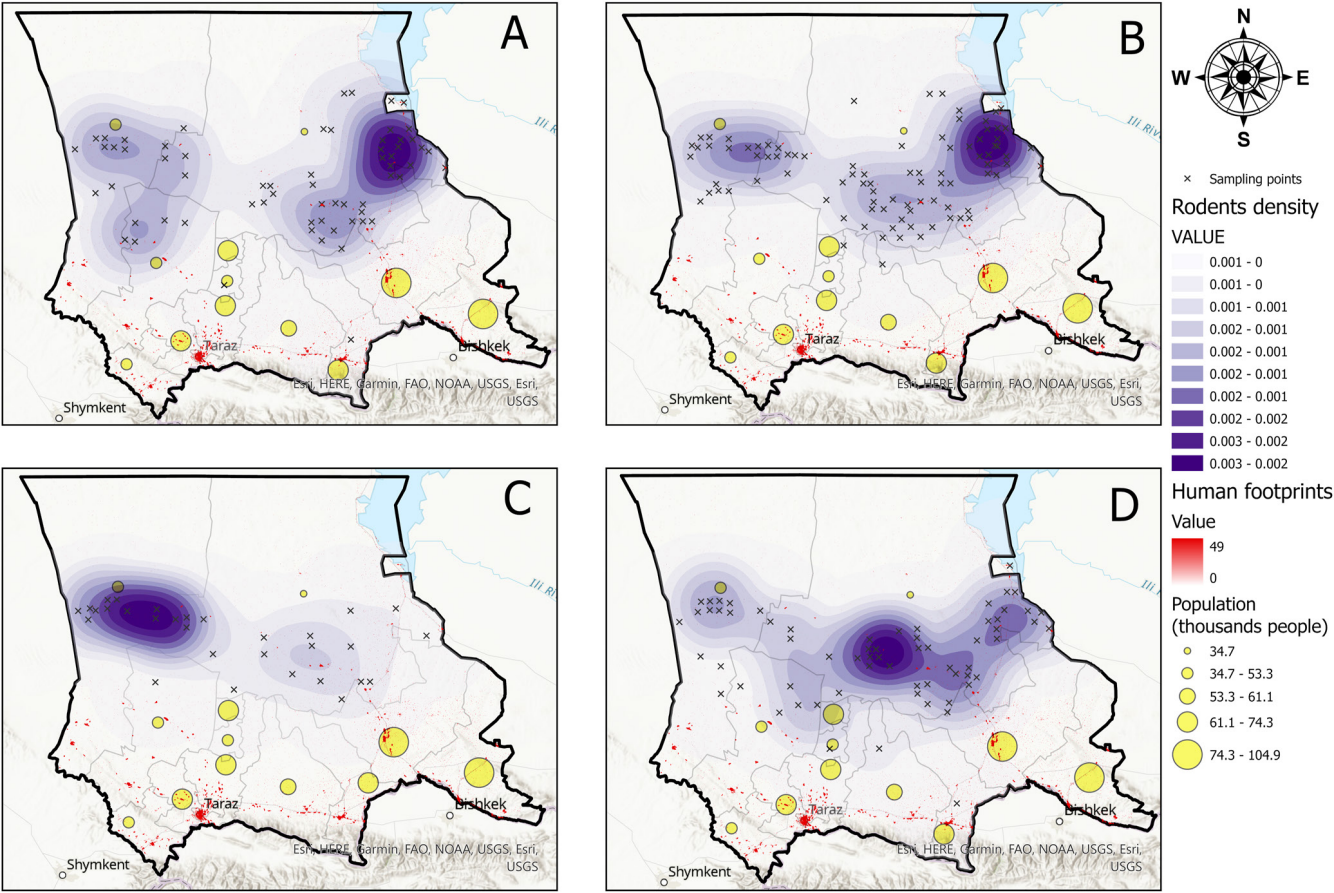
Rodent and human population densities. (a) Libyan jird (*Meriones lybicus*), (b) Great gerbil (*Rhombomys opimus*), (c) Yellow ground squirrel (*Spermophilus fulvus*), (d) Midday jird (*Meriones meridianus*). Note: The crosses indicate sampling points. The yellow circles show the human population sizes. Human footprints (red) represent resources and energy quantities consumed and produced by humans during their lifetime.

We created Species distribution Forecasts (SDFs) primarily aimed at predicting the future distribution of four rodent species, specifically for the plague risk areas in the southern part of Kazakhstan for the year 2100. For these future predictions, we utilized three different climate scenarios. The SDFs for both the total rodent plague hotspots and the ones with the AB records have been presented in Figures 4–13.

Comparing the distribution hotspots in Figures 4a and 4b, we can observe that in comparison with the distribution of rodents without antibodies, the distribution of rodents carrying antibodies is significantly smaller, yet more concentrated in the central area, along Moyinqum Desert.

In Figure 5, we can observe reverse patterns as for Figure 4 in regards to total and AB models. The distribution of rodents carrying plague antibodies is significantly larger than those individuals not carrying them.

Compared to the situation from the year 2000, for the MIROC scenario, we can observe that the high‐intensity hotspots of rodents with no antibodies will expand and concentrate along the Moyinqum Desert, with the overall distribution of these samples decreasing in the northern part of the Zhambyl region. Conversely, the high‐intensity hotspots of rodents with AB records will decrease in the central part of Moyinqum, yet evenly distributes to the north of the Zhambyl region and south of the Qaragandy region.

Compared to the situation from the year 2000, presented in Figure 4, for the MRI scenario in Figure 6, we can observe that the hotpots for species not carrying antibodies significantly decrease in the north of the Zhambyl region. The high‐intensity hotspot for rodents carrying antibodies is smaller for the modeled distribution in 2100 using this MRI scenario compared to the year 2000. The distribution of these samples increases insignificantly toward the north (Qaragandy region) and northeast (Almaty region).

Once more, the pattern we noticed in previous figures is present here. In 2100 under the IPSL scenario, the hotspots of species with antibodies are significantly smaller, while the hotspots of those without antibodies are much larger, compared to the year 2000. Additionally, the distribution range of species with plague antibodies has increased, while the range of species without antibodies has decreased compared to the distribution in 2000. A more detailed description of the results can be found in Text S1 in Supporting Information S1.

## 4 Discussion

Over the past 30 years, the area of natural plague foci in Kazakhstan has increased significantly and currently exceeds 1,117,000 km^2^, which is approximately 41% of the country's territory. There are seven natural and 15 autonomous foci in the country, within which more than 90 landscape epizootological regions have been identified (Abdel et al., 2021; Atshabar et al., 2015). The various natural centers of plague in Kazakhstan differ in size, intensity of the disease's spread, level of available scientific knowledge of the ecosystem and spatial structures, and risk of epidemiological complications. The manifestation of plague among wild animals occurs continuously in varying degrees of intensity and locations, and with different intervals between outbreaks.

### 4.1 Species Richness and SDM of Total Plague Risk Individuals

Species richness represents the number of different species occupying a particular ecological niche, or region (Moore, 2013). In our case, we analyzed four rodent species—great gerbil, yellow ground squirrel, Libyan jird, and midday jird—presenting the main plague risk in the Zhambyl region, and created the combined SDMs (Figures 2, 3, 4, 5, 6, 7). With the limited data from the mentioned region, we analyzed other species of small mammals, possibly carrying the infection, and created combined and individual SDMs based on species with or without AB records (see Figures S1–S17 in Supporting Information S1). We also created the SDMs for species with unavailable AB records (Figures S16 and S17 in Supporting Information S1). The distribution of rodents and other small mammals was examined in the south and south‐eastern parts of Kazakhstan, and while the data have been collected from the Zhambyl region, the generated models showed spill‐overs into neighboring regions.

As seen by the SDMs of the main risk species (Figures S7–S10 in Supporting Information S1), the Moiynqum, Betpaqdala, and Tauqum areas represent the natural focal hotspots. This is supported by our own estimation (Figure 8). The vegetation in the area is characteristic of the Kazakh Steppe desert and semi‐desert areas, mostly consisting of shrub and grass species; such habitats create favorable conditions for populations of rodents and other small mammals carrying the plague (Biodiversity Assessment for Kazakhstan, 2001). The areas with higher occurrences of considered rodents, in particular, the great gerbil, represent a higher total plague risk.

As seen in Figure 8, the greatest number of local residents (shown in red spots) in the Zhambyl region is concentrated in the southern cities and towns, such as Taraz, Shu, Karatau, and Korday, while the highest population density of the four rodent species studied is located in the central area of the region, in particular, Betpaqdala Desert to the west, Moiynqum Desert in the center, and Tauqum Desert in the eastern part of the region. This is supported by the SDMs shown in Figures 2 and 3, where the hotspots occur throughout the central part of the Zhambyl region, including the two largest districts in the region—Moiynqum and Sarysu, with approximately 30,000 local residents. This means that the highest risk of plague is directed toward the local population in the rural areas, towns, and villages.

For the neighboring Almaty region, created SDMs suggest that the rodents mostly occur in the Balkhash district in the western part and the Ile district in the eastern part of species' distribution. With the highly populated rural areas such as Baqanas in the Balkhash district, and Otegen‐Batyr in the Ile district, the contact with the population of rodents possibly carrying the plague should be kept under control. The spread of the plague commonly begins with the contact of local residents with the carriers of the plague—the infected flea vector. It is also common where domestic animals (Oyston & Williamson, 2011) and livestock, in particular camels (Abdeliyev et al., 2022) were first infected and then transmitted the plague to humans. Plague can also be transmitted from the human and animal corpses and carcasses, in particular, by direct (by skin) and indirect (by inhalation) contact with their blood and sputum (Jullien et al., 2021).

Our findings highlight the urgent need for adequate preventative measures and policies aimed at reducing the risk of the spread of plague. To that end, local populations would benefit from in‐depth education aimed at reducing human‐wildlife contact, along the following points: (a) proper handling of personal protective equipment when dealing with human and animal corpses and carcasses; (b) vaccinating the livestock and companion animals against plague; (c) minimizing contact with any wild animals and wearing protective clothing in the areas where the plague could be circulating. Considering past fatal outcomes and present and future risks, educating local populations is the priority and a low‐cost strategy for reducing the risk of plague transmission.

### 4.2 Movement Forecasts of Plague Risk Species

The shifts in chemical composition throughout the lower atmosphere of the Earth, as well as substantial alterations in chemical composition in its upper atmosphere (Kirk‐Davidoff, 2018; Shah et al., 2020), and subsequent continuous change of the climate, are one of the factors that affect biodiversity and the human population. It is especially relevant to analyze the possible changes in the occurrences of small mammals possibly carrying the plague. Based on the results obtained, we can expect an increase or decrease in possible plague risk based on the different climate scenarios and create the foundation for future plague management.

Comparing the present distribution models in Figures 2, 3, 4, we can observe that, overall, the small mammals are most abundant in the central area of the Zhambyl region, in particular, in the Moiynqum Deserts, throughout the Moiynqum and Sarysu districts. The hotspots are also present in the Tauqum Massif in the Almaty region.

In the case of low temperature increase or cooling MIROC scenario of climate change in 2100, the distribution hotspot of small mammals with total plague risk, shown in Figure 5, centralizes in the Moiynqum Deserts and, in the case of AB‐positive samples, increases in the Betpaqdala in the north area of Zhambyl region and the south of the Qaragandy region, continuing northward. On the contrary, according to the most likely MRI scenario of a temperature increase of 2 degrees Celsius, the distribution range of species with no AB record, shown in Figure 6, will centralize in Moiynqum and seem to expand into the eastern direction—throughout the Tauqum Massif and the south‐east side of Balkhash Lake. Lastly, similarly to the MIROC scenario, in the IPSL scenario of a major temperature increase of 3 degrees Celsius, the model in Figure 7, shows high‐intensity hotspots in the Moyinqum Deserts, and for the AB‐positive samples, an expansion of the distribution range in the Zhambyl region toward the north and the east in 2100.

Overall, it can be seen that the distribution ranges of the species studied will expand throughout two districts of the Zhambyl region with the highest population density– Moiynqum and Sarysu districts—and move northwards and eastwards toward the Qaragandy and Almaty regions. The latter is one of the regions with the largest populations in Kazakhstan. With expected future climate change, the plague risk will thus likely increase exponentially. The heightened risk of a plague outbreak could lead to declines in wildlife and human populations, as well as potential health and economic impacts for the region and thus health. Since currently animal and human mortality for plague are not actively recorded for Kazakhstan and other central Asian countries, heightened control of plague risk and management of wildlife and human population health is necessary.

In our analysis, we can observe that for all current and future scenarios, the AB‐positive distribution was smaller and less widespread compared to the total sampled group. In most cases, it seems like the AB‐positive hotspots can be observed in the center of the total distribution hotspots. This indicates that the centers of these total distribution hotspots have likely been infected with the plague before and have built up antibodies over time. Considering the disease risks, it appears that the outer bounds of the modeled total distribution can be considered to carry the highest disease risk. The reason is that in those outer bounds, the disease infection risks are high, but with very low AB presence, thereby putting many species at risk to be infected.

In light of the evidence, it would be appropriate for public‐health measures to be adopted in order to minimize the risk of plague transmission to humans in the area. This includes increased monitoring and surveillance of wildlife populations for signs of disease, as well as educational campaigns to raise awareness among the local populace about the dangers of close contact with wildlife. Additionally, it may be necessary to implement measures to control the populations of the species in question or to implement quarantine measures during possible plague outbreaks to prevent the spread of the disease. Further research and studies are crucial to determine the most effective measures to prevent the increase of plague risk in the Zhambyl region and in the rest of the nation.

## 5 Conclusion

In this study, we investigated the spatial distribution of the rodents that present a threat for plague (*Y*. *pestis*) transmission and, consequently, ascertained the areas where the plague risk could be prevalent in southern Kazakhstan. By using Super Species Distribution Modeling, we determined that the rodents possibly carrying the plague are most abundant in the Moiynqum and Betpaqdala Deserts in the Zhambyl region and the Tauqum Massif in the Almaty region, indicating these areas as hotspots for natural foci of the disease. The Zhambyl region's central area, including the Moiynqum and Sarysu districts, exhibits numerous hotspots, indicating a high potential risk of plague outbreak for the rural areas, towns, and villages. Meanwhile, in the neighboring Almaty region, the distribution of rodents that potentially carry the disease is mainly concentrated in the Balkhash and Ile districts. Due to the high population in rural areas such as Baqanas and Otegen‐Batyr, which are situated in these districts, it is essential to regulate the interaction between the local population and rodents. With future climate change scenarios, the plague risk is likely to increase in these areas, which could lead to declines in local wildlife, as well as potential health and economic impacts for the region and the world. Measures, including increased monitoring and surveillance of wildlife populations, and campaigns for educating the public about reducing interaction with wildlife (especially, with the great gerbil, Libyan jird, midday jird, and yellow ground squirrel), are necessary to minimize the risk of plague transmission to humans in the area. Further research is needed to determine the most effective measures to prevent the increase of plague risk in the Zhambyl region and in Kazakhstan in general.

## Conflict of Interest

The authors declare no conflicts of interest relevant to this study.

## Supporting information

Supporting Information S1

## Data Availability

The spatial data used for modeling MaxEnt, Spatial analysis and mapping in the study are available at Zenodo via (Rametov et al., 2023) with a Creative Commons Attribution 4.0 International license.
