# Supplementary material for: Mapping Plague Risk Using Super Species Distribution Models and Forecasts for Rodents in the Zhambyl Region, Kazakhstan
Source: Geohealth. 2023 Nov 13;7(11):e2023GH000853. doi: 10.1029/2023GH000853 (PMC10641984; doi:10.1029/2023GH000853)
Supplement: Supplementary file 1 — Supporting Information S1 [file GH2-7-e2023GH000853-s001.docx]

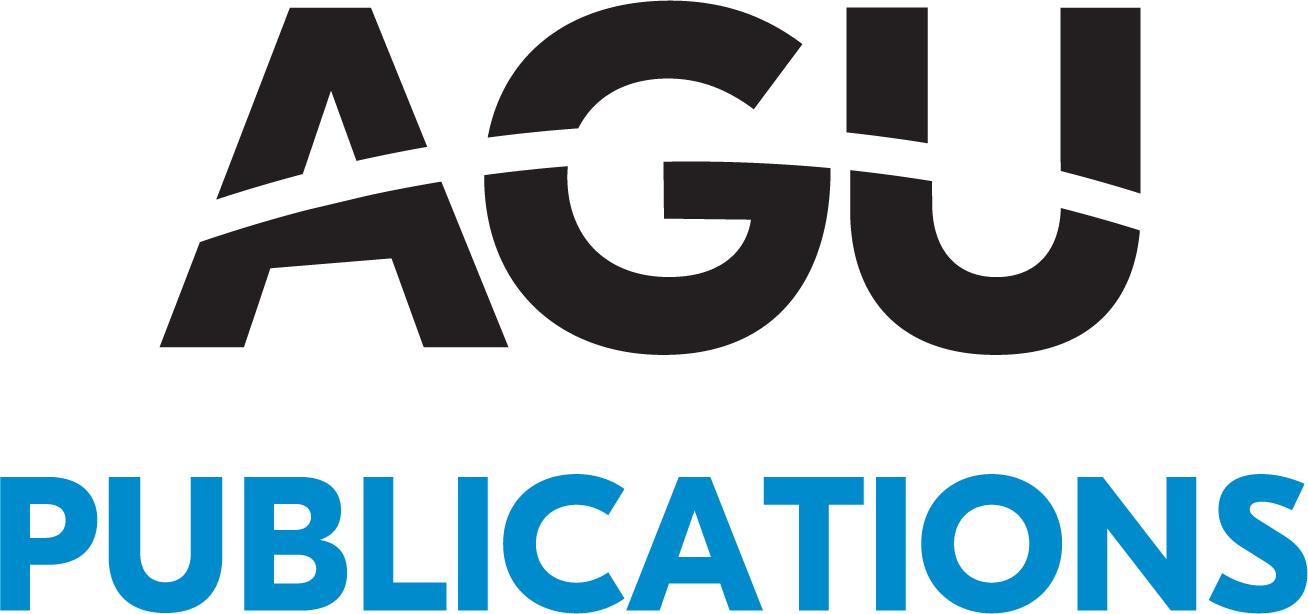


*GeoHealth*

Supporting Information for

**Mapping Plague risk using Super Species Distribution Models and Forecasts for Rodents in the Zhambyl region, Kazakhstan**

N.M. Rametov^1,2,3^, M. Steiner^4^†, N.A. Bizhanova^5,6,7^†, Z.Zh. Abdel^1^†, D.T. Yessimseit^1^, B.Z. Abdeliyev^1^, R.S. Mussagalieva^1^

† These authors have contributed equally to this work and share first authorship

^1^National Scientific Center for Particularly Dangerous Infections named after M. Aikimbaev, Almaty 050054, Kazakhstan; 1, 4, 5, 6, 7.

^2^Institute of Ionosphere, Almaty 050020, Kazakhstan; 1.

^3^Department of Geospatial Engineering, Satpaev Kazakh National Research Technical University, Almaty 050000, Kazakhstan; [n.rametov@stud.satbayev.university](mailto:n.rametov@stud.satbayev.university); 1.

^4^Department of Animal Science, Wageningen University and Research, Wageningen 6708 PB, The Netherlands; [moriz.steiner@wur.nl](mailto:moriz.steiner@wur.nl); 2.

^5^Laboratory of Theriology, Institute of Zoology, Almaty 050060, Kazakhstan; [nazerke.bizhanova@zool.kz](mailto:nazerke.bizhanova@zool.kz); 3.

^6^Department of Biodiversity and Bioresources, Al-Farabi Kazakh National University, Almaty 050040, Kazakhstan; 3.

^7^Wildlife Without Borders Public Fund, Almaty 050063, Kazakhstan; 3.

Corresponding author: Nurkuisa Rametov ([nurkuisa.rametov@gmail.com](mailto:nurkuisa.rametov@gmail.com))

**Contents of this file**

Text S1

Figures S1 to S17

Tables S1 to S3

**Introduction**

The following supporting information includes additional information such as sampling records (Tables S1 and S3), top predictors used for creating Species Distribution Models (SDMs) (Table S2), SDMs created combining all nine observed host small mammal species (Figures S1-S6) and for all included species individually (Figures S7-S17), as well as the detailed analysis of the species distribution in the region (Text S1).

**Text S1. Detailed Results discussion**

As seen in Figure 2, the total plague risk in the south of Kazakhstan is predominantly concentrated in the central area of the Zhambyl region. In particular, the highest occurrences of the four studied rodent species can be traced in the west of Moiynqum, which is a natural focus of plague. To the west of the Sarysu district of the Zhambyl region, the hotspots occur in the Suzak district of the Turkestan region, also located in the Moiynqum Desert area, to the left riverside of the Shu River. The observed hotspots move in the direction from the west into the Moiynqum Desert in the Zhambyl region to the east into the Tauqum Massif in the Almaty region, another natural plague focus. Located in the Balkhash-Alakol Basin, the Tauqum populations of considered rodents mostly occupy the areas to the east and east-south of Balkhash Lake and Ile River, with the occurrences decreasing in the southern and eastern areas around the Bozoi Plateau.

The species contributing the most to the SDM shown in Figures 2 and S7 is the great gerbil (*Rhombomys opimus*). As illustrated in the SDM in Figure S7a, this rodent is most abundant in the western area of Moiynqum Desert, the southern area of Betpaqdala Desert, and in the south and south-east shores of the Balkhash Lake, with some occurrences present in the Tauqum Massif.

Similarly to the great gerbil, hotspots of the species such as the Libyan jird (*Meriones libycus*), midday jird (*Meriones meridianus*), and yellow ground squirrel (*Spermophilus fulvus*) (Figures S8a, S9a and S10a, respectively, see Appendix C) can also be observed in the western and central areas of Moiynqum and around the southern shores of Balkhash Lake.

Regarding other species of small mammals presenting less plague risk, a similar distribution tendency can be traced for the small five-toed jerboa (*Allactaga elater*) (Figure S11a). The Tamarisk jird (*Meriones tamariscinus*) (Figure S17a) can also be observed to have hotspots in the Moiynqum area. The distribution of representatives of the Family *Soricidae* (Figure S15a) is notable, as we can see their higher occurrence not only along the Moiynqum and Betpaqdala in the north of Zhambyl region but also throughout all areas around the south of the Balkhash Lake, thus “embracing” this lake area. According to the SDMs, other shrews, such as the lesser white-toothed shrew and other representatives of the genus *Crocidura* (Figures S16c and S16d, respectively), as well as the great dwarf hamster (Figure S16b) and house mouse (*Mus musculus*) (Figure S17c) are highly abundant throughout Moiynqum, Betpaqdala, Tauqum, and Western Tien Shan near the border of Kazakhstan, Kyrgyzstan, and Uzbekistan. The distribution of the lesser white-toothed shrew, great dwarf hamster, and house mouse is also prominent in the southeastern area of the Balkhash Lake. The distribution of long-tailed marmot (*Marmota caudata*) (Figure S16f) is notable, as the hotspots can be observed in the Betpaqdala Desert area, throughout the southern shore of the Balkhash Lake towards Tauqum, Northern Tien Shan, the Zhetisu Alatau Mountain ranges in the south and south-east, and towards Western Tien Shan and partially Central Tien Shan in the border region of Kazakhstan and Kyrgyzstan. As shown in these models, Balkhash Lake is essential for the survival of these and other species in the region.

The steppe polecat (*Mustela eversmanii*), on the other hand, is less associated with the Balkhash Lake. According to the generated model, shown in Figure S14a), this mustelid is most abundant in the western and eastern sides of Moiynqum, with hotspots being in the border area between the Sarysu district of the Zhambyl region, as well as the Suzak district of Turkestan region, and in the east of Moiynqum district. We can additionally note its occurrence in the Tauqum Massif and Bozoi Plateau in the Almaty region. To the south of Tauqum is the course of the Ile River, with ‘toqai’, – tree-shrub vegetation on its banks providing sufficient cover for the steppe polecat and its prey base, one of which is the great gerbil, also inhabiting this area. The distribution of other mustelids, such as the least weasel (*Mustela nivalis*) and marbled polecat (*Vormela peregusna*) (Figures S17d and S17e, respectively) is less mosaic, with the hotspots located along Moiynqum and around the south-eastern shore of the Balkhash Lake.

Rodents such as the Wood mouse (*Apodemus sylvaticus*) (Figure S12a) and common vole (*Microtus arvalis*) (Figure S13a) have hotspots in the borders between Kazakhstan and Kyrgyzstan, covering the majority of the Kyrgyz Alatau Mountains of Northern Tien Shan and the eastern area of Western Tien Shan. The same distribution pattern in the mentioned region can be noted for the species with no AB record such as the Evermann’s hamster (*Allocricetulus eversmanni*), tolai hare *(Lepus tolai*), and social vole (*Microtus socialis)* (Figures S16a, S16e and S17b, respectively). The vegetation cover in both of these mountain regions is dominated by juniper shrubs (*Juniperus pseudosabina, J. sibirica*), creating optimal conditions for hiding and nesting for these and other species of animals inhabiting the area.


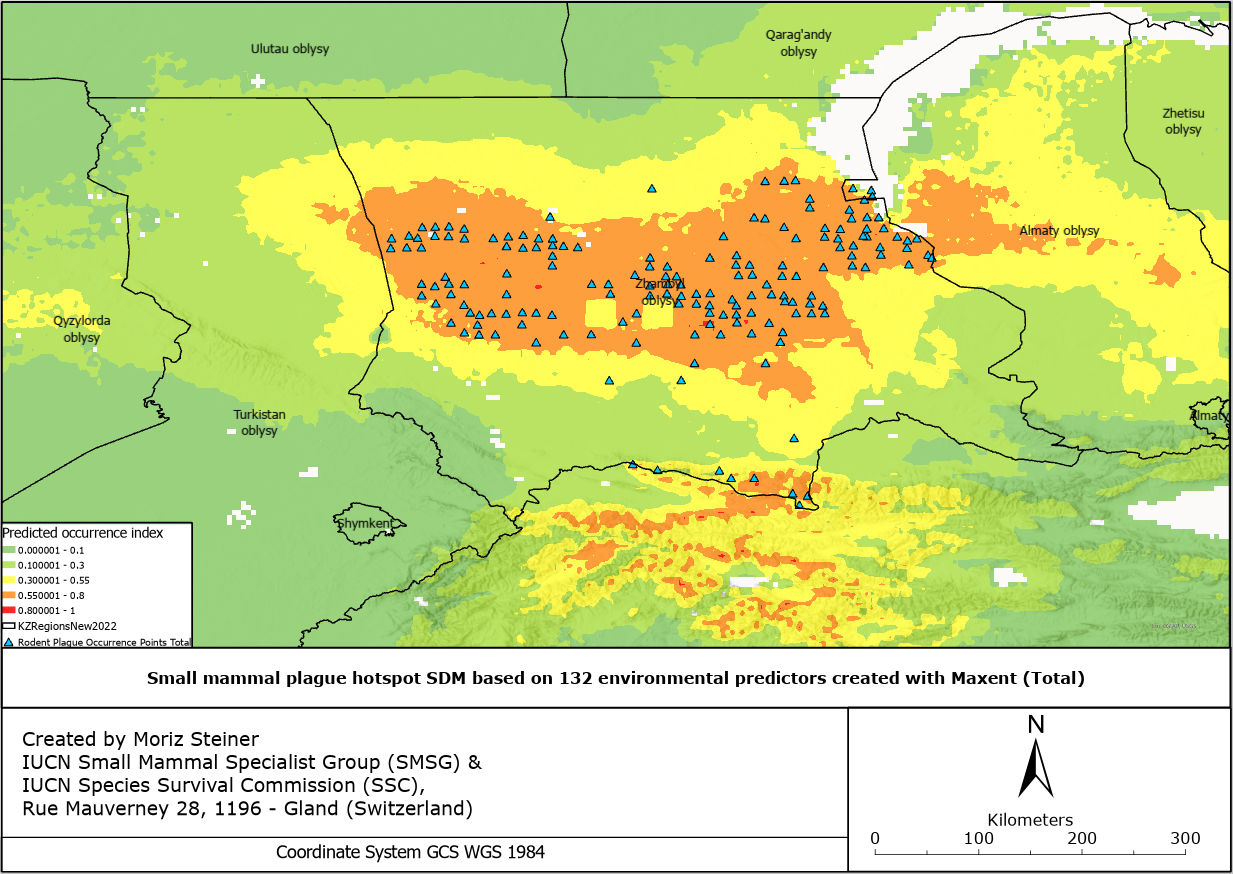


**Figure S1.** Plague hotspot SDM based on 132 environmental predictors based on nine species of small mammals (Total)


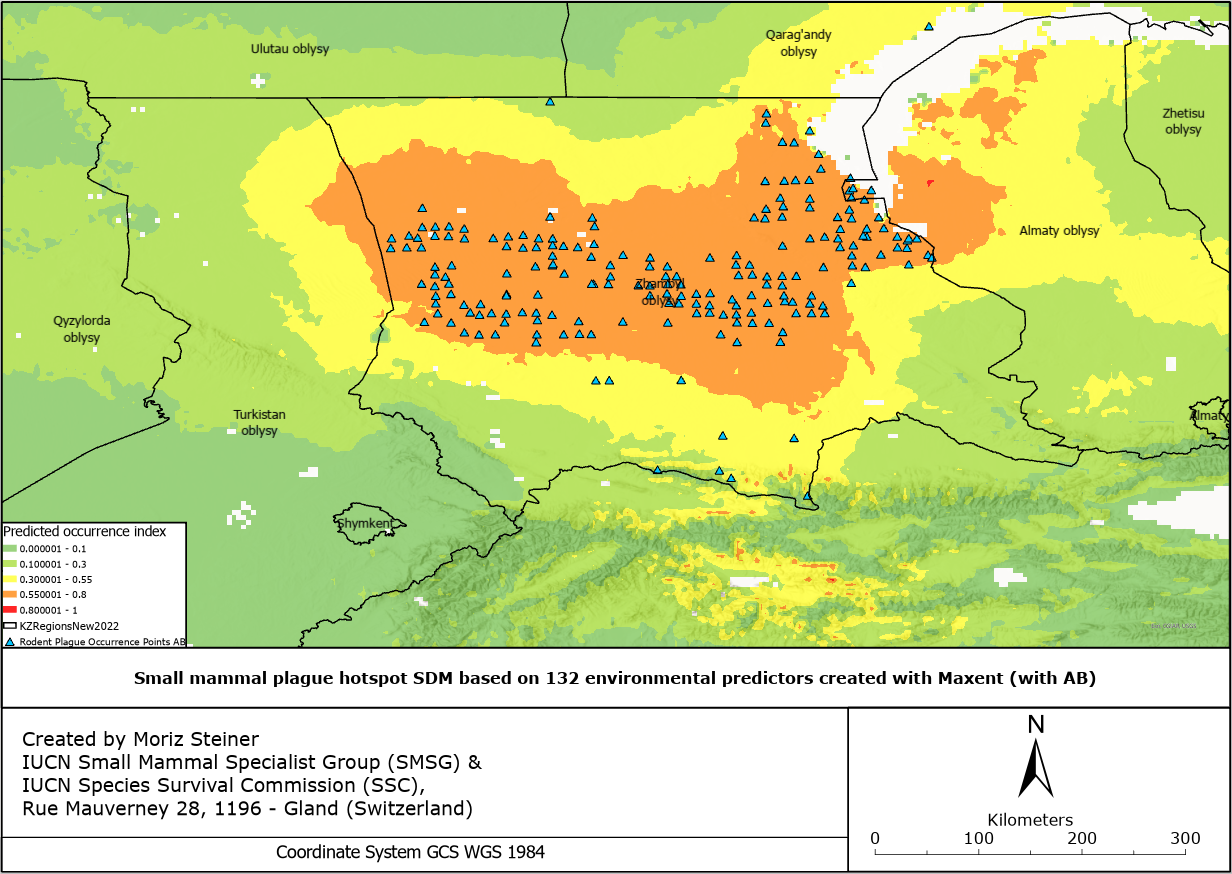


**Figure S2.** Plague hotspot SDM based on 132 environmental predictors based on nine species of small mammals (with AB)


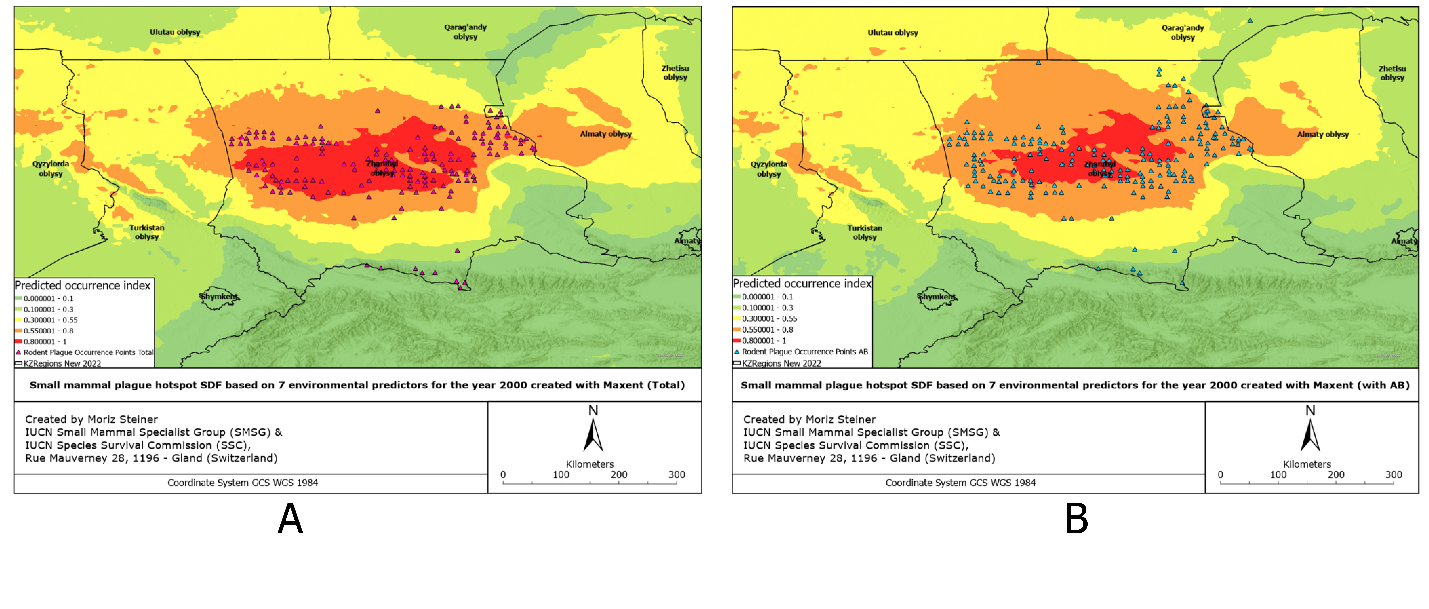


**Figure S3.** Small mammal plague hotspot SDF based on 7 environmental predictors for the year 2000 created with Maxent a) Total, b) with AB


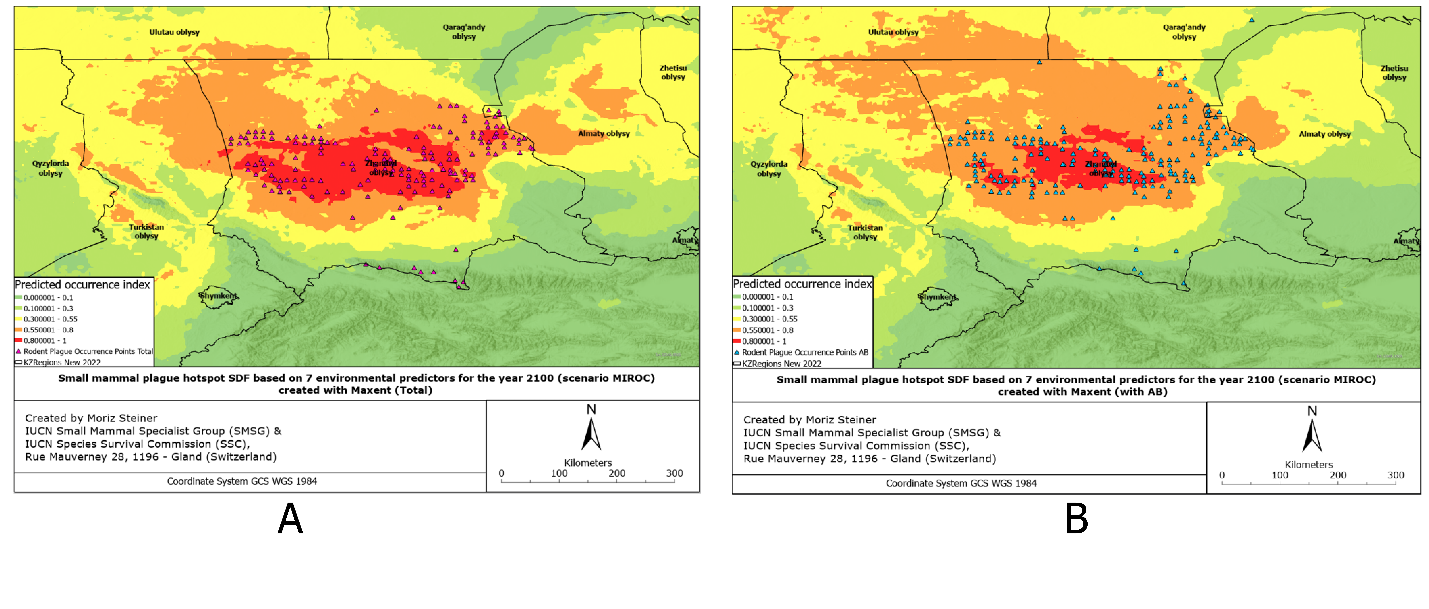


**Figure S4.** Small mammal plague hotspot SDF based on 7 environmental predictors for the year 2100 (scenario MIROC) created with Maxent a) Total, b) with AB


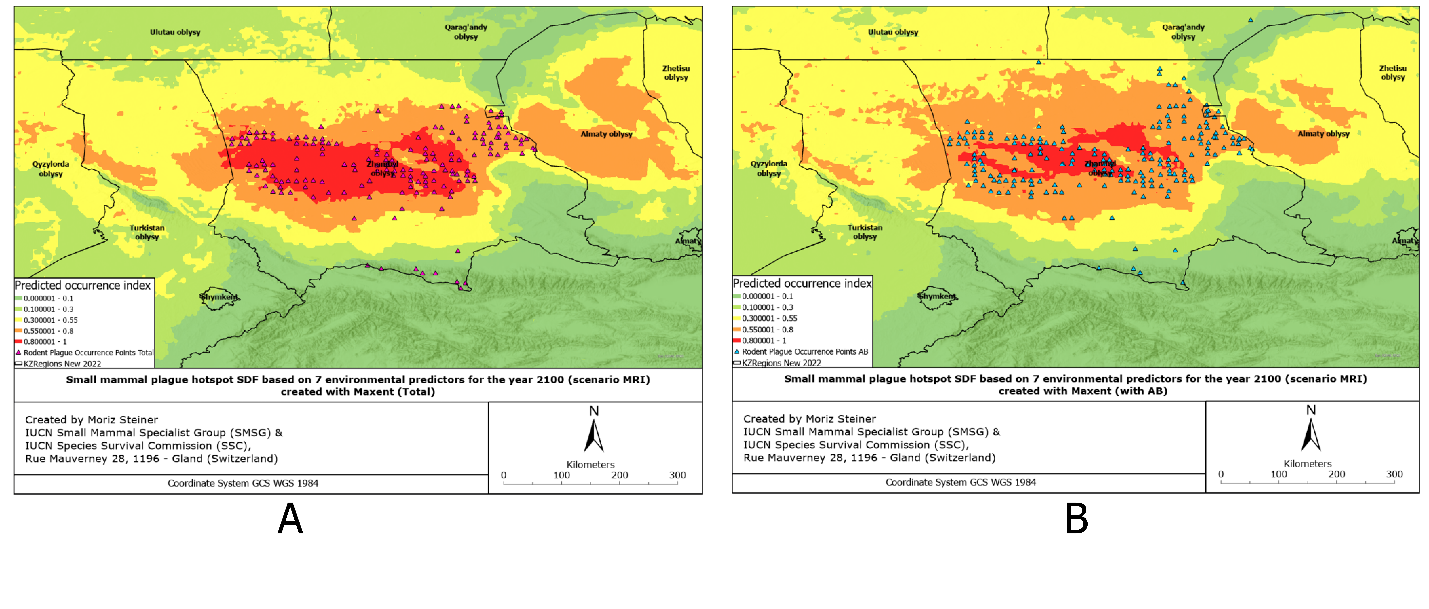


**Figure S5.** Small mammal plague hotspot SDF based on 7 environmental predictors for the year 2100 (scenario MRI) created with Maxent a) Total, b) with AB


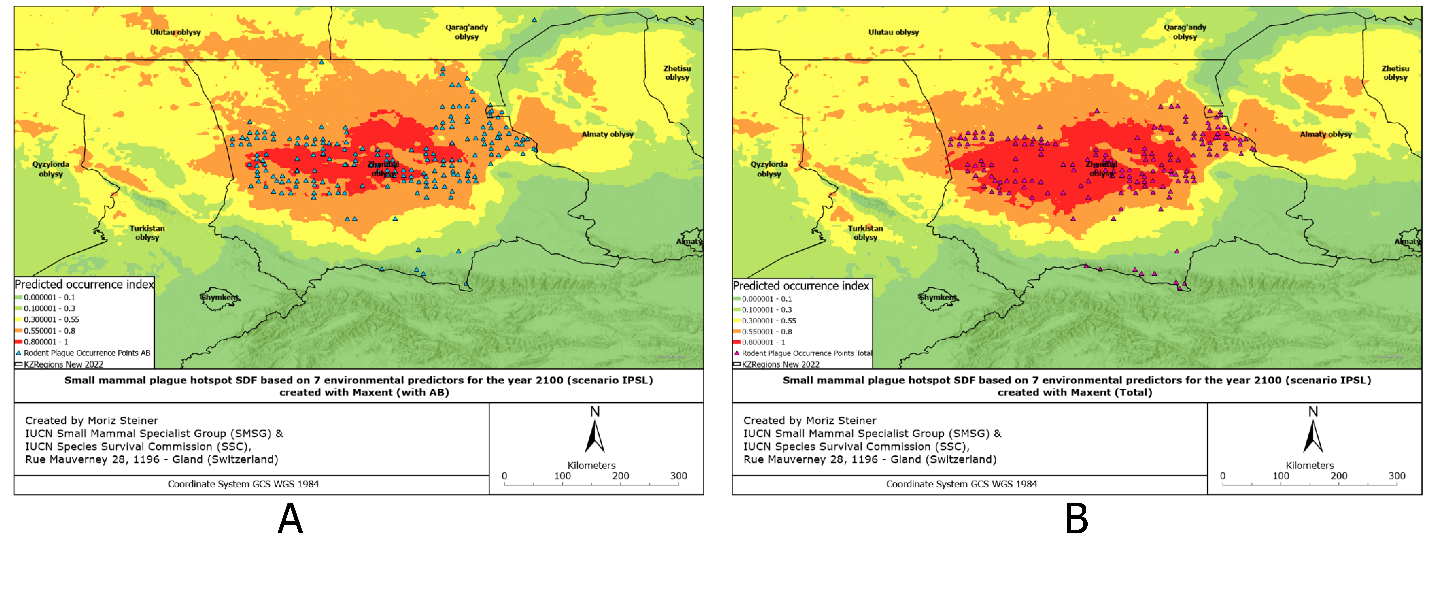


**Figure S6.** Small mammal plague hotspot SDF based on 7 environmental predictors for the year 2100 (scenario IPSL) created with Maxent a) Total, b) with AB


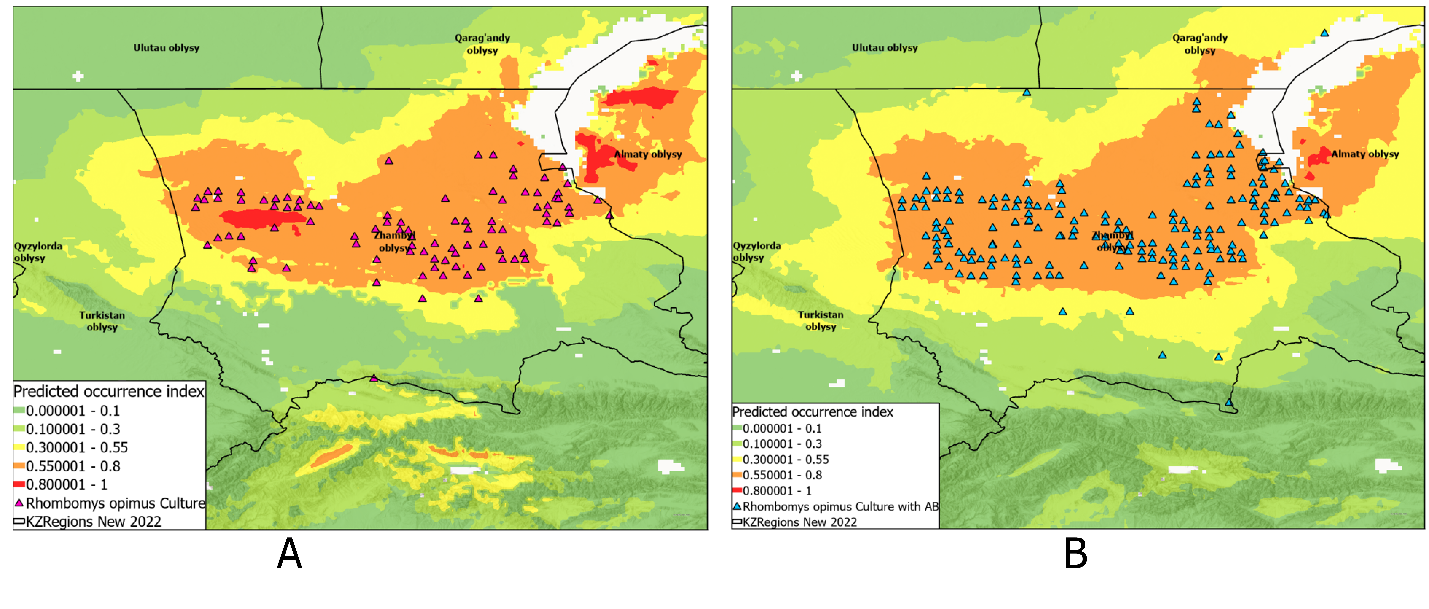


**Figure S7.** Great gerbil (*Rhombomys opimus*) SDM based on 132 environmental predictors created with Maxent a) (Culture), b) (Culture with AB)


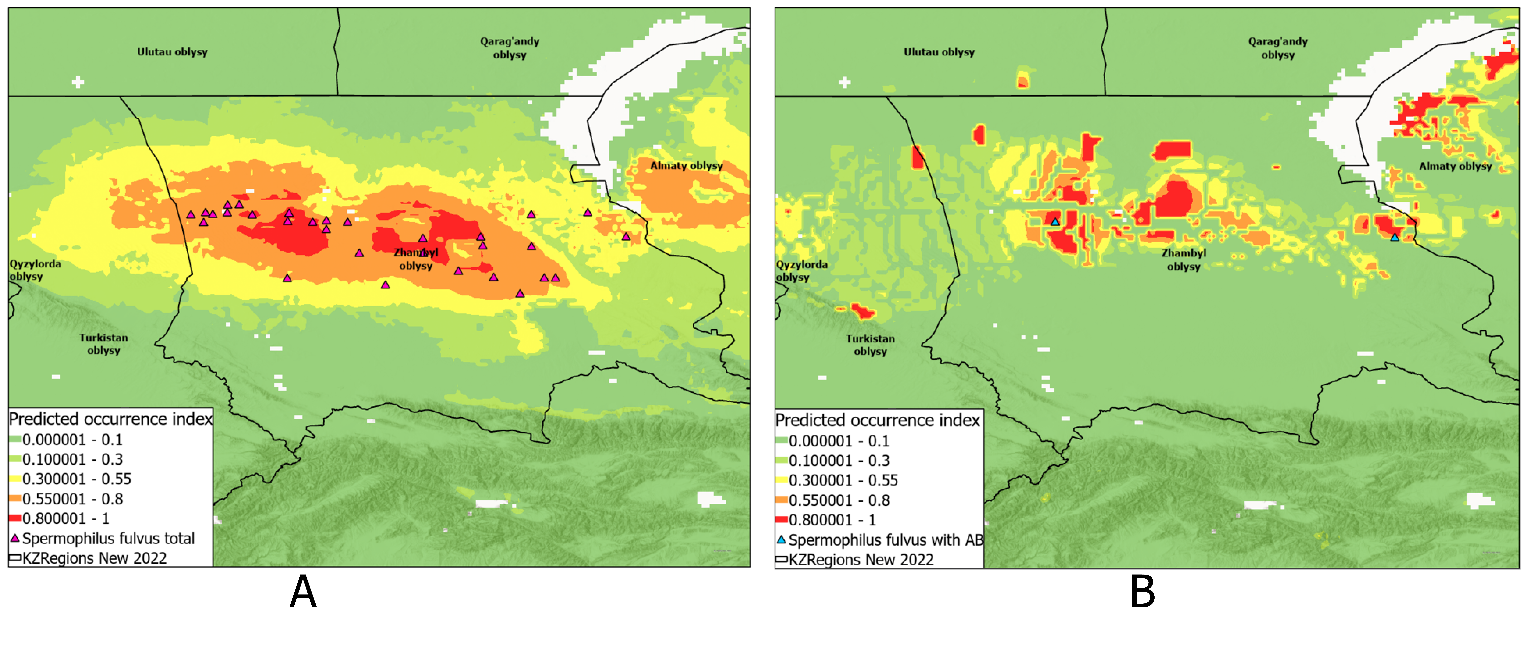


**Figure S8.** Yellow ground squirrel (*Spermophilus fulvus*) SDM based on 132 environmental predictors created with Maxent a) (total), b) (with AB)


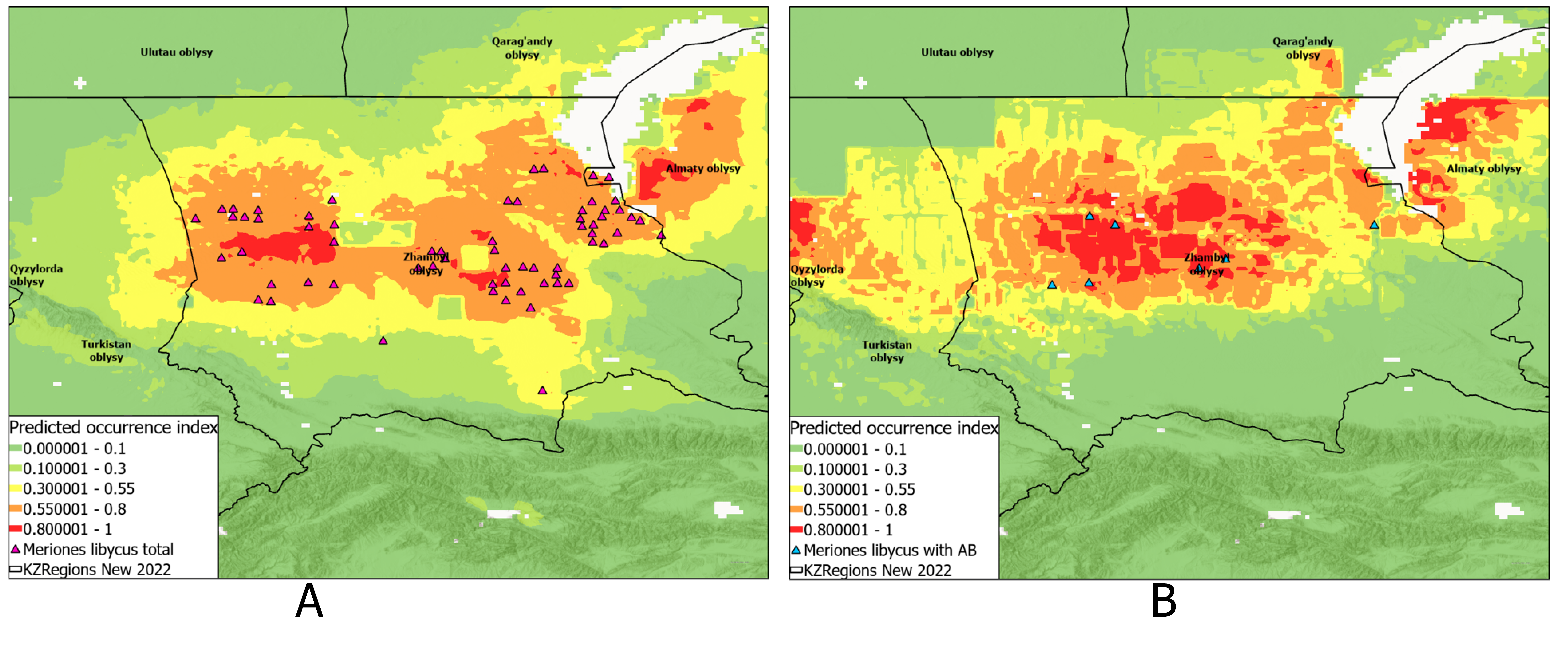


**Figure S9.** Libyan jird (*Meriones libycus*) SDM based on 132 environmental predictors created with Maxent a) (total), b) (with AB)


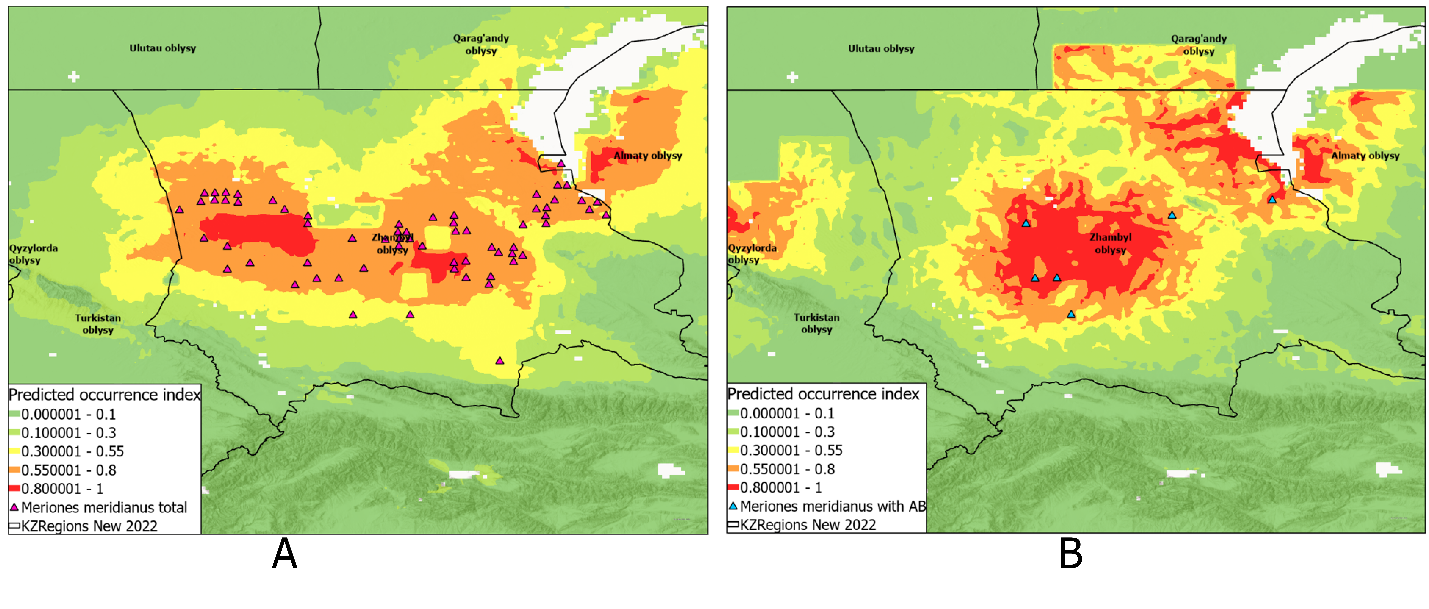


**Figure S10.** Midday jird (*Meriones meridianus*) SDM based on 132 environmental predictors created with Maxent a) (total), b) (with AB)


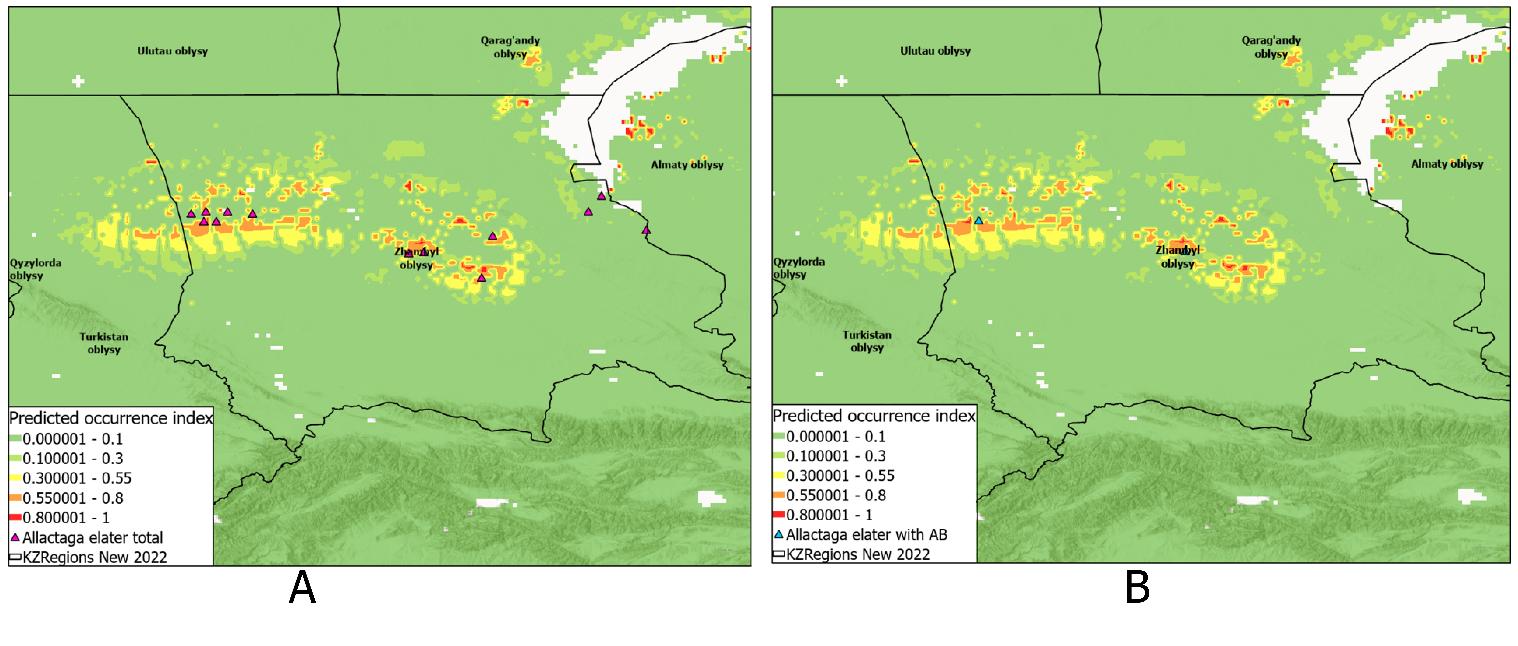


**Figure S11.** Small five-toed jerboa (*Allactaga elater*) SDM based on 132 environmental predictors created with Maxent a) (total), b) (with AB)


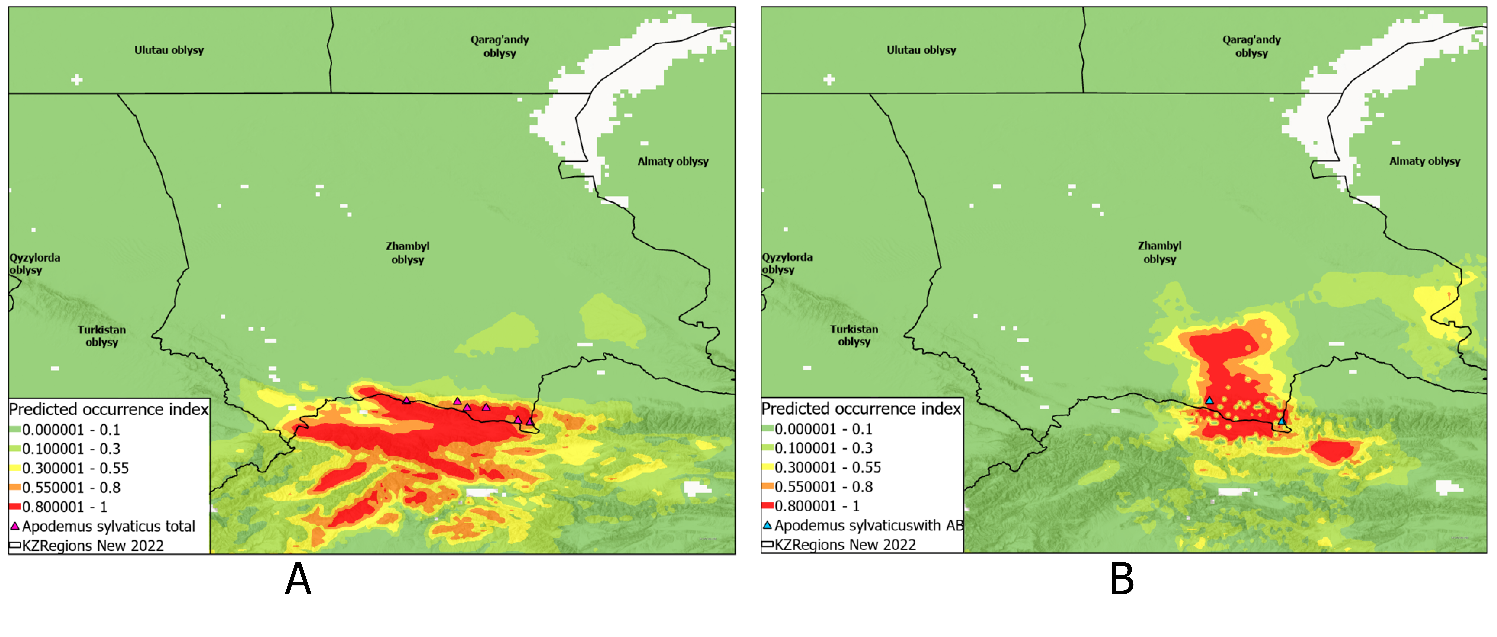


**Figure S12.** Wood mouse (*Apodemus sylvaticus*) SDM based on 132 environmental predictors created with Maxent a) (total), b) (with AB)


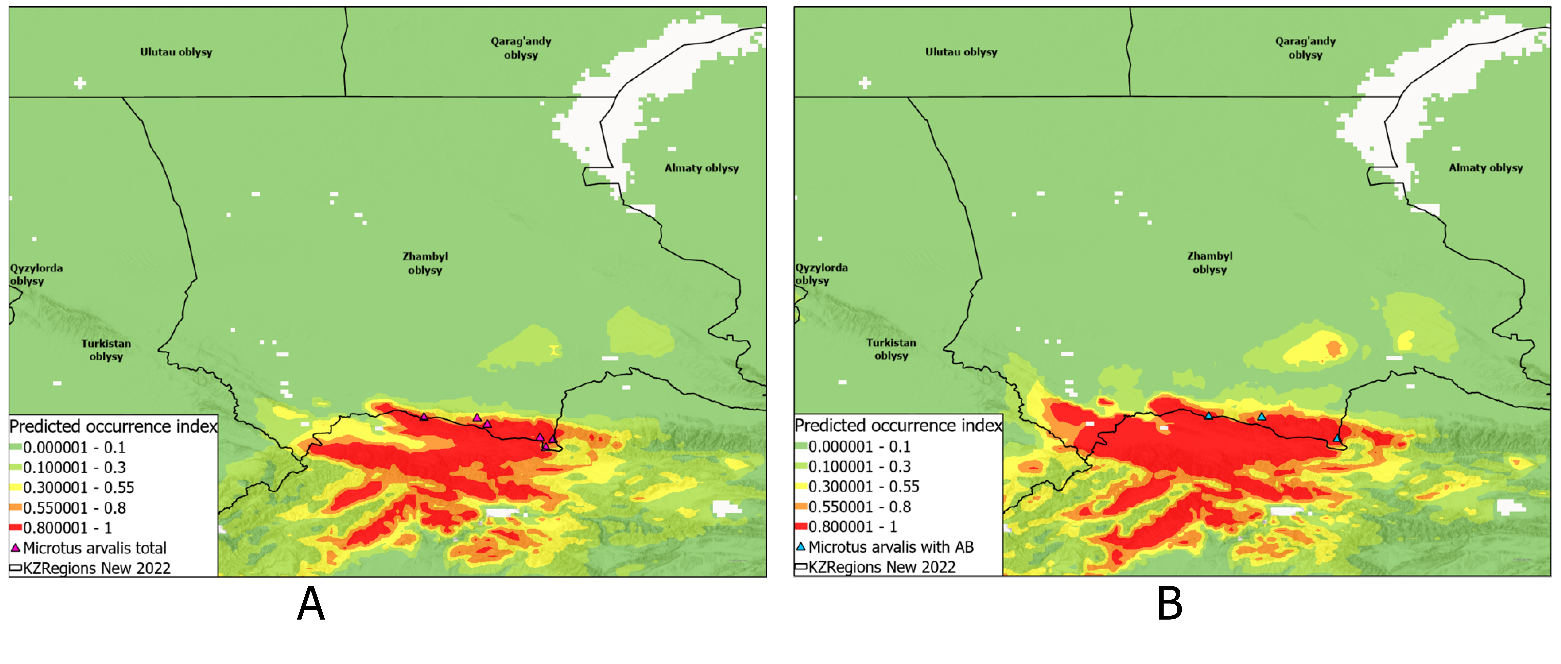


**Figure S13.** Common vole (*Microtus arvalis*) SDM based on 132 environmental predictors created with Maxent a) (total), b) (with AB)


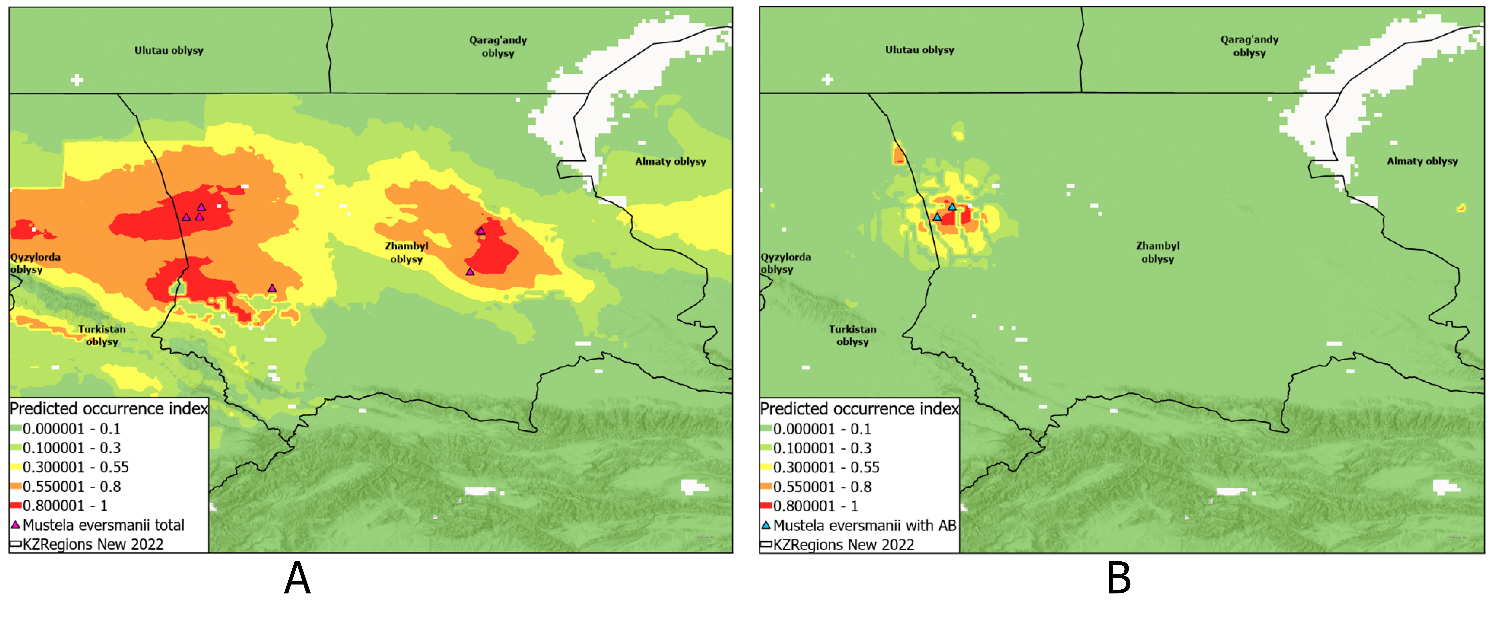


**Figure S14.** Steppe polecat (*Mustela eversmanii*) SDM based on 132 environmental predictors created with Maxent a) (total), b) (with AB)


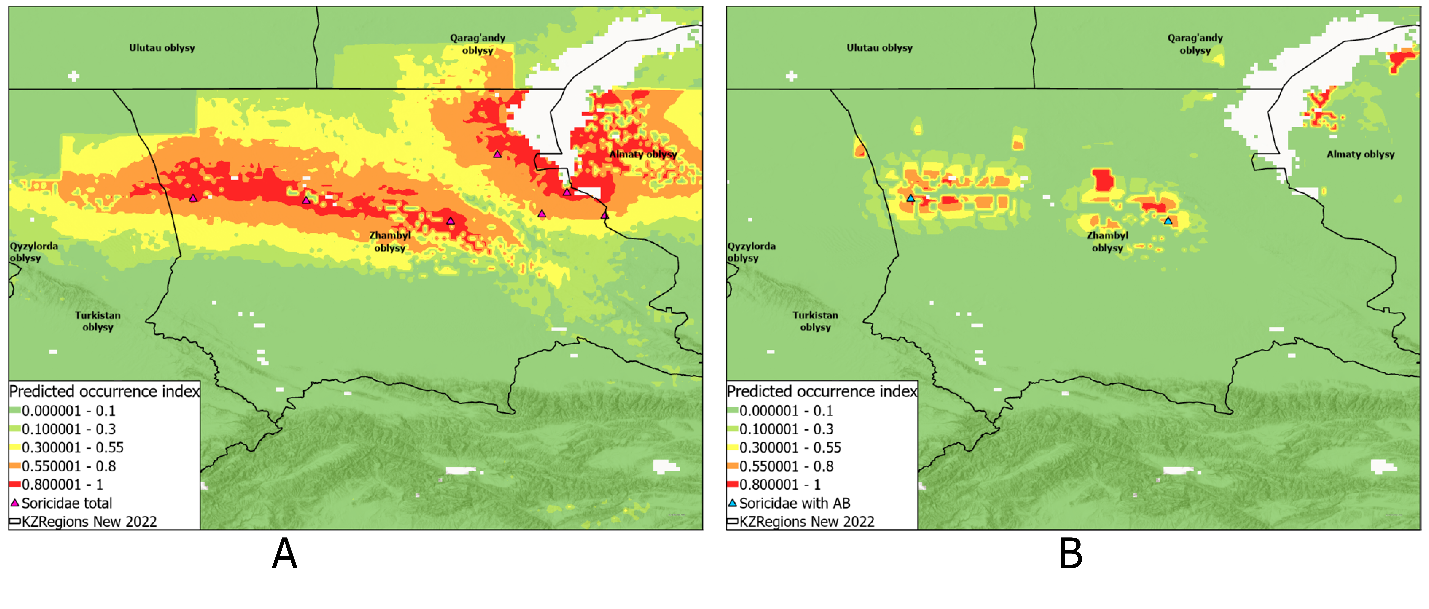


**Figure S15.** Shrews (*Soricidae*) SDM based on 132 environmental predictors created with Maxent a) (total), b) (with AB)


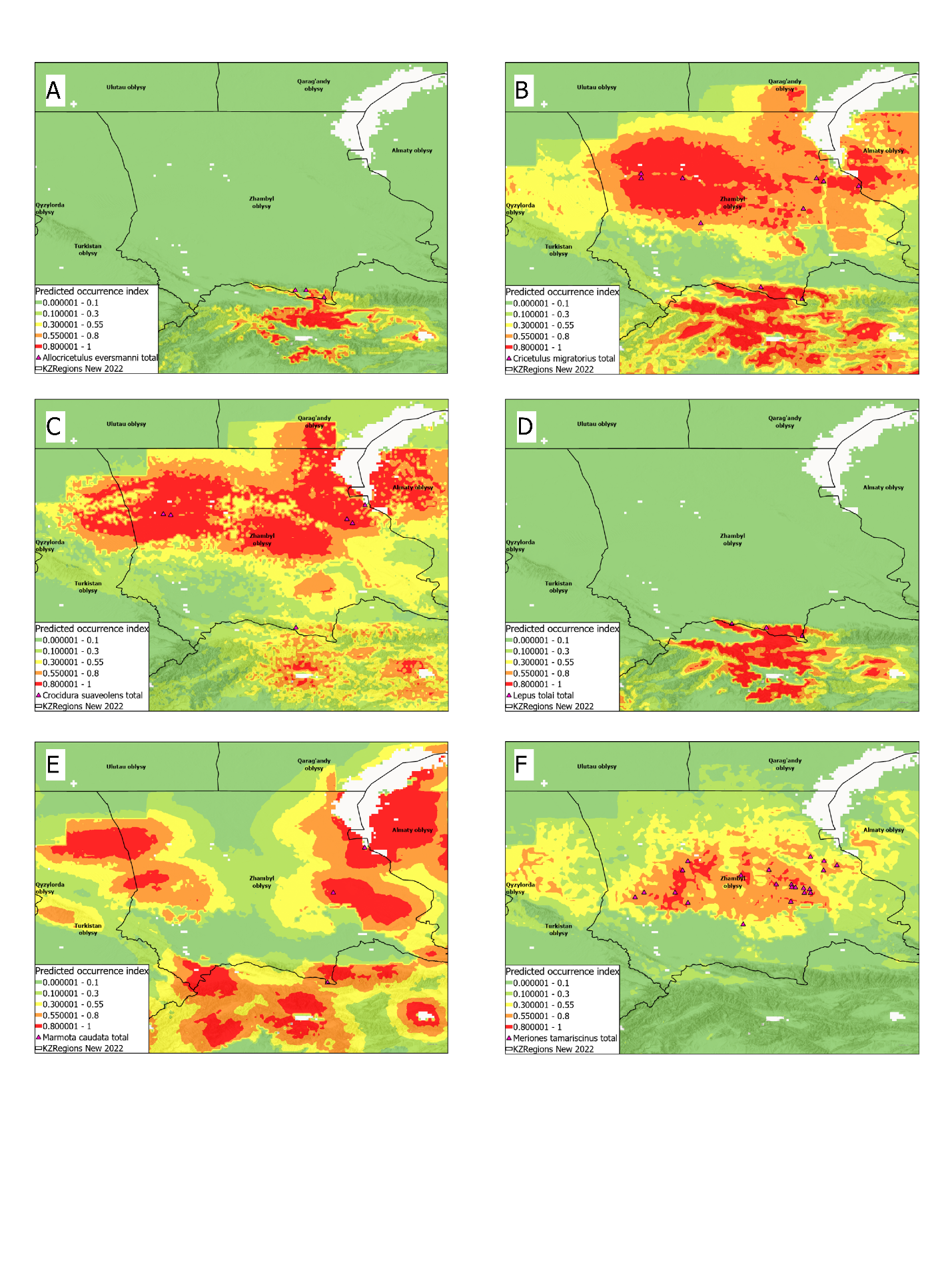


**Figure S16.** SDM of species with unavailable AB records based on 132 environmental predictors created with Maxent a) Eversmann’s hamster (Allocricetulus eversmanni), b) grey dwarf hamster (Cricetulus migratorius), c) lesser white-toothed shrew (Crocidura suaveolens), d) white-toothed shrews (Crocidura), e) tolai hare (Lepus tolai), f) long-tailed marmot (Marmota caudata).


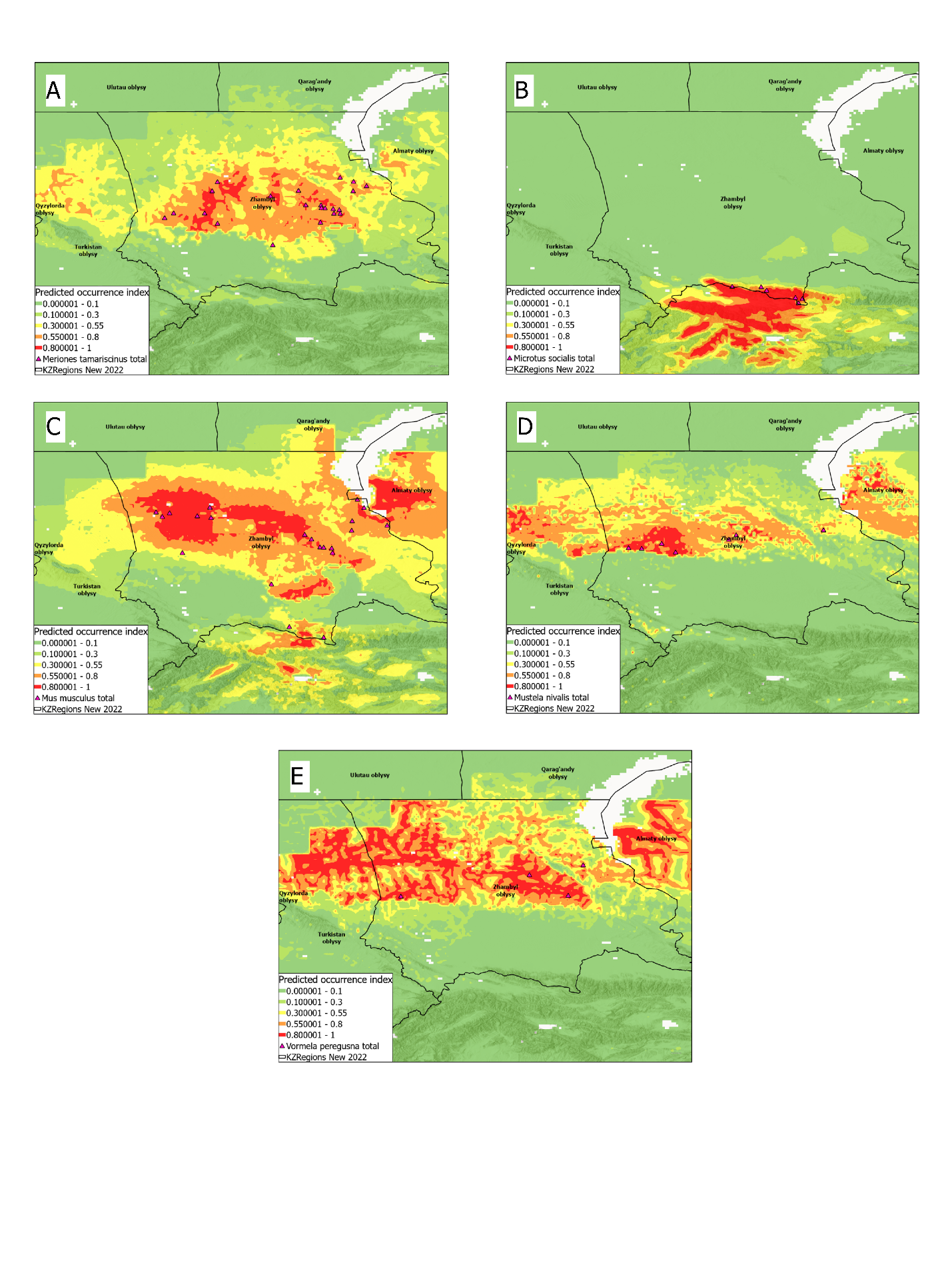


**Figure S17.** SDM of species with unavailable AB records based on 132 environmental predictors created with Maxent a) Tamarisk jird (*Meriones tamariscinus*), b) social vole (*Microtus socialis*), c) house mouse (*Mus musculus*), d) least weasel (*Mustela nivalis*), e) marbled polecat (*Vormela peregusna*)

| Years | Great gerbil (*Rhombomys opimus*) | Libyan jird (*Meriones lybicus*) | Midday jird (*Meriones meridianus*) | Yellow  ground squirrel (*Spermophilus fulvus*) | Other species  (*n* = 18) | Sampling (*n*) | AB positive (*n*) | Culture (*n*) | Positive | |
| --- | --- | --- | --- | --- | --- | --- | --- | --- | --- | --- |
|  |  |  |  |  |  |  |  |  | *n* | % |
| 2000 | 1894 | 156 | 186 | 28 | 185 | 2449 | 298 | 13 | 311 | 12.70 |
| 2001 | 2145 | 76 | 125 | 14 | 142 | 2502 | 259 | 6 | 265 | 10.59 |
| 2002 | 2675 | 132 | 179 | 9 | 162 | 3157 | 186 | 3 | 189 | 5.99 |
| 2003 | 4675 | 58 | 312 | 27 | 476 | 5548 | 292 | 22 | 314 | 5.66 |
| 2004 | 4367 | 243 | 134 | 21 | 358 | 5123 | 412 | 9 | 421 | 8.22 |
| 2005 | 3756 | 145 | 245 | 28 | 583 | 4757 | 73 | 10 | 83 | 1.74 |
| 2006 | 3923 | 22 | 0 | 4 | 1069 | 5018 | 174 | 4 | 178 | 3.55 |
| 2007 | 4959 | 36 | 250 | 12 | 679 | 5936 | 128 | 7 | 135 | 2.27 |
| 2008 | 11631 | 34 | 342 | 9 | 591 | 12607 | 169 | 21 | 190 | 1.51 |
| 2009 | 7477 | 86 | 272 | 2 | 634 | 8471 | 195 | 3 | 198 | 2.34 |
| 2010 | 8344 | 97 | 310 | 24 | 640 | 9415 | 158 | 13 | 171 | 1.82 |
| 2011 | 8454 | 208 | 258 | 11 | 253 | 9184 | 242 | 28 | 270 | 2.94 |
| 2012 | 7909 | 134 | 212 | 11 | 338 | 8604 | 103 | 15 | 118 | 1.37 |
| 2013 | 8982 | 36 | 125 | 4 | 425 | 9572 | 68 | 14 | 82 | 0.86 |
| 2014 | 8608 | 147 | 123 | 8 | 415 | 9301 | 34 | 22 | 56 | 0.60 |
| 2015 | 8590 | 181 | 200 | 7 | 261 | 9239 | 9 | 3 | 12 | 0.13 |
| 2016 | 7939 | 217 | 207 | 5 | 695 | 9063 | 12 | 6 | 18 | 0.20 |
| 2017 | 7677 | 160 | 305 | 5 | 393 | 8540 | 15 | 1 | 16 | 0.19 |
| 2018 | 6154 | 61 | 97 | 13 | 322 | 6647 | 10 | 0 | 10 | 0.15 |
| 2019 | 5832 | 45 | 94 | 29 | 422 | 6422 | 22 | 0 | 22 | 0.34 |
| 2020 | 5690 | 45 | 78 | 11 | 322 | 6146 | 13 | 0 | 13 | 0.21 |
| Total | 142948 | 2438 | 4122 | 291 | 9365 | 160002 | 2875 | 200 | 3075 | 3.02 |

**Table S1.** Sampling records and antibody (AB) confirmation, by years (2000-2020).

| Predictor name | Source | Explanation | Citation |
| --- | --- | --- | --- |
| BIO1_2_5min - BIO19_2_5min; tmin1 - tmin12; tmax1 - tmax12; tavg1 - tavg12; srad1 - srad12; prec1 - prec12; Wcaltitude | Worldclim (<https://www.worldclim.org/data/worldclim21.html>) | These datasets represent most of the climate data utilized for the SDM. | Fick and Hijmans, 2017 |
| FAOCC | FAO Geonetwork (<http://www.fao.org/geonetwork/>) | This predictor represents the global climate classes. |  |
| LC12asc2; VE4 | Geospatial Information Authority of Japan (<https://www.gsi.go.jp/kankyochiri/gm_global_e.html>) | These predictors represent the global land cover (LC12asc2), and the global vegetation cover (VE4). |  |
| GlobalRiversProxy2 | A Simple Global River Bank full Width & Depth Database (<http://gaia.geosci.unc.edu/rivers/>) | This predictor represents all global mid-size and large rivers. | Andreadis et al., 2013 |
| GlobalBigRivers11 | Global major rivers (<https://www.arcgis.com/home/item.html?id=44e8358cf83a4b43bc863646cd695945>) | This predictor represents all global large rivers. |  |
| GlobalCities2 | Global Cities (<https://hub.arcgis.com/datasets/6996f03a1b364dbab4008d99380370ed_0?geometry=-65.394%2C25.931%2C73.737%2C49.818>) | This predictor represents all global cities. |  |
| GlobalLakes2 | Global Lakes and Wetlands Database (GLWD) (<http://www.fao.org/land-water/land/land-governance/land-resources-planning-toolbox/category/details/es/c/1043160/>) | This predictor represents all global lakes and wetlands. |  |
| GlobalSnowCoverMonthJan2021_7; FFJan2020_3; FFFeb2020_3; FFMar2020_3; FFMay2020_3, FFJun2020_3; FFJul2020_3; FFAug2020_3; FFSep2020_3; FFOct2020_3; FFNov2020_3; FFJan2021_3 | Global Snow Cover and Forest fires (https://neo.sci.gsfc.nasa.gov/view.php?datasetId=MOD10C1_M_SNOW and <https://neo.sci.gsfc.nasa.gov/view.php?datasetId=MOD14A1_M_FIRE&year=2020>) | These predictors mainly represent the Global snow cover in the month of January, and the forest fire information for nearly all months of 2020 with exception of April and December as these months were not available. |  |

**Table S2.** Environmental predictors.

| Predictor name | Source | Explanation | Citation |
| --- | --- | --- | --- |
| WorldSoil2 | Global Soil characteristics map (<https://webarchive.iiasa.ac.at/Research/LUC/External-World-soil-database/HTML/HWSD_Data.html?sb=4>) | This predictor represents all global soil types and its characteristics. |  |
| WorldProtectedAreasMerged4 | Global Protected areas. (<https://www.protectedplanet.net/en/search-areas?geo_type=region&filters%5Bis_type%5D%5B%5D=terrestrial>) | This predictor represents all global protected areas merged into one shapefile. | UNEP-WCMC and IUCN, 2020 |
| WorldMammaldensity4; WorldRodentDensity3; WorldThreatenedMammalDensity3; GlobalBirdDensity2 | Global Mammal density. Proximity maps for the world mammal density, world rodent density, world threatened mammal density (<https://biodiversitymapping.org/index.php/mammals/>) | These predictors mainly represent global biodiversity densities. In detail, they contain the world mammal density, world rodent density, world bird density, and the world's threatened mammal density. | Jenkins et al., 2013; Pimm et al., 2014 |
| GlobalRoadsProxy2 | Global Roads - Socioeconomic data and applications center (SEDAC) - Data center in NASA's Earth Observatory System Data and Information System (EOSDIS) (<https://sedac.ciesin.columbia.edu/data/set/groads-global-roads-open-access-v1/data-download>) | This predictor represents the global proximity to all world's roads. Minor roads may not be included. |  |
| HII1 | Human Influence Index (HII). (<https://sedac.ciesin.columbia.edu/data/set/wildareas-v2-human-influence-index-geographic/data-download>) | This predictor represents the global Human Influence index |  |
| WorldSlope1 | Slope. (<https://scholarworks.alaska.edu/handle/11122/7151>) | This predictor represents the global terrestrial and aquatic slope. | Sriram and Huettmann, 2017 |
| World_MAX_RH_JAN - World_MAX_RH_DEC; World_MIN_RH_JAN - World_MIN_RH_DEC | Global Monthly Relative Humidity. (<http://palebludata.com/?q=data>) | This predictor set represents the global maximum and minimum relative humidity for the months January to December of the year 2020. | Jones and Wint, 2015 |

**Continuation of Table S2.** Environmental predictors.

| Order | Family | Genus | Species | TSN | Records type |
| --- | --- | --- | --- | --- | --- |
| Eulipotyphla | Soricidae |  |  |  | Total, AB |
|  |  | Crocidura |  |  | Total |
|  |  |  | Lesser white-toothed shrew  (*Crocidura suaveolens*) |  | Total |
| Carnivora | Mustelidae | Mustela | Least weasel  (*Mustela nivalis*) |  | Total |
|  |  |  | Steppe polecat  (*Mustela eversmanii*) | 726280 | Total, AB |
|  |  | Vormela | Marbled polecat  (*Vormela peregusna*) |  | Total |
| Lagomorpha | Leporidae | Lepus | Tolai hare  (*Lepus tolai*) |  | Total |
| Rodentia | Sciuridae | Spermophilus | Yellow ground squirrel  (*Spermophilus fulvus*) | 632449 | Total, AB, Culture |
|  |  | Marmota | Long-tailed marmot  (*Marmota caudata*) |  | Total |
|  | Dipodidae | Allactaga | Small five-toed jerboa  (*Allactaga elater*) | 609735 | Total, AB |
|  | Cricetidae | Cricetulus | Grey dwarf hamster  (*Cricetulus migratorius*) |  | Total |
|  |  | Allocricetulus | Eversmann’s hamster  (*Allocricetulus eversmanni*) |  | Total |
|  |  | Rhombomys | Great gerbil  (*Rhombomys opimus*) | 632995 | Culture, Culture with AB |
|  |  | Microtus | Social vole  (*Microtus socialis*) |  | Total |
|  |  |  | Common vole  (*Microtus arvalis*) | 632856 | Total, AB |
|  | Muridae | Meriones | Libyan jird  (*Meriones libycus*) | 632980 | Total, AB, Culture |
|  |  |  | Midday jird  (*Meriones meridianus*) | 632981 | Total, AB, Culture |
|  |  |  | Tamarisk jird  (*Meriones tamariscinus*) |  | Total |
|  |  | Mus | House mouse  (*Mus musculus*) |  | Total |
|  |  | Apodemus | Wood mouse  (*Apodemus sylvaticus*) | 585145 | Total, AB |

**Table S3.** Complete list of species with field data records.
